# Endoplasmic reticulum stress and mitochondrial injury are critical molecular drivers of AlCl_3_-induced testicular and epididymal distortion and dysfunction: protective role of taurine

**DOI:** 10.1007/s00418-022-02111-2

**Published:** 2022-05-05

**Authors:** Hanaa A. Khalaf, Ayman Z. Elsamanoudy, Salwa M. Abo-Elkhair, Fatma E. Hassan, Passant M. Mohie, Fatma M. Ghoneim

**Affiliations:** 1 Medical Histology and Cell Biology Department, Faculty of Medicine, Mansoura University, Mansoura, 35516 Egypt; 2 Medical Biochemistry and Molecular Biology Department, Faculty of Medicine, Mansoura University, Mansoura, 35516 Egypt; 3 Clinical Biochemistry Department, Faculty of Medicine, King Abdulaziz University, Jeddah, 21465 Saudi Arabia; 4 Medical Physiology Department, Kasr Alainy, Faculty of Medicine, Cairo University, Cairo, Egypt; 5 Clinical Pharmacology Department, Faculty of Medicine, Alexandria University, Alexandria, Egypt

**Keywords:** Taurine, Testes, ER stress, Mitochondrial injury, PCNA, Vimentin

## Abstract

Aluminum, the third most plentiful metal in the Earth’s crust, has potential for human exposure and harm. Oxidative stress plays an essential role in producing male infertility by inducing defects in sperm functions. We aimed to investigate the role of endoplasmic reticulum (ER) stress and mitochondrial injury in the pathogenesis of aluminum chloride (AlCl_3_)-induced testicular and epididymal damage at the histological, biochemical, and molecular levels, and to assess the potential protective role of taurine. Forty-eight adult male albino rats were separated into four groups (12 in each): negative control, positive control, AlCl_3_, and AlCl_3_ plus taurine groups. Testes and epididymis were dissected. Histological and immunohistochemical (Bax and vimentin) studies were carried out. Gene expression of *vimentin*, *PCNA*, *CHOP*, *Bcl-2*, *Bax*, and *XBP1* were investigated via quantitative real-time polymerase chain reaction (qRT-PCR), besides estimation of malondialdehyde (MDA) and total antioxidant capacity (TAC). Light and electron microscopic examinations of the testes and epididymis revealed pathological changes emphasizing both mitochondrial injury and ER stress in the AlCl_3_ group. Taurine-treated rats showed a noticeable improvement in the testicular and epididymal ultrastructure. Moreover, they exhibited increased gene expression of *vimentin*, *Bcl-2*, and *PNCA* accompanied by decreased *CHOP*, *Bax*, and *XBP1* gene expression. In conclusion, male reproductive impairment is a significant hazard associated with AlCl_3_ exposure. Both ER stress and mitochondrial impairment are critical mechanisms of the deterioration in the testes and epididymis induced by AlCl_3_, but taurine can amend this.

## Introduction

Aluminum (Al) is an extensively disseminated metal in the environment and can thus potentially cause toxic damage to different human tissues and organs (Shaw 2018). Al compounds are widely used in medicines such as antacids, vaccines, antidiarrheals, phosphate binders, and allergen injections (Igbokwe et al. 2019), food additives, toothpaste (Saad et al. 2018), and water purification agents (Kalaiselvi et al. 2014).

It has been described that aluminum chloride (AlCl_3_) produces reactive oxygen species (ROS) (Al-Otaibi et al. 2018) and inhibits antioxidant enzymes in the blood, seminal plasma, testis, liver, kidney, lung, and brain (Güvenç et al. 2020). Excessive production of ROS causes oxidative stress in spermatozoa, producing male infertility (Agarwal and Sengupta 2020). AlCl_3_-induced toxicity in the epididymis, vas deferens, seminal vesicle, and ventral prostate of mice was also reported by ALMurshidi et al. (2021). Moreover, exposure to AlCl_3_ has been reported to affect testicular development and testosterone synthesis in experimental animals (Lokman et al. 2021).

Xu et al. (2017) found that AlCl_3_ exposure promotes mitochondrial oxidative stress, which may contribute to mitochondrial energy metabolism disorder and liver dysfunction. Testicular mitochondria regulate different aspects of male reproductive functions, such as spermatogenesis, adenosine triphosphate (ATP) synthesis, and Leydig cell steroidogenesis (Ibrahim et al. 2019). In addition, sperm mitochondria are involved in many essential fertility processes, such as sperm motility, hyperactivation, capacitation, and acrosome reaction (Durairajanayagam et al. 2021).

Furthermore, it was reported that AlCl_3_ exposure induces endoplasmic reticulum (ER) stress in rat brain (Promyo et al. 2020). Under specific conditions, ER stress can be beneficial to the body; however, if ER protein homeostasis is not restored, the prolonged activation of the unfolded protein response (UPR) may initiate apoptotic cell death via upregulation of the CCAAT enhancer-binding protein (C/EBP) homologous protein (CHOP). CHOP induces apoptosis via suppression of the prosurvival protein B-cell lymphoma-2 (Bcl-2). Bcl-2-associated X (Bax) was also upregulated during ER stress in cardiomyocyte models (Wang et al. 2018). Li et al. (2014) showed that CHOP-deficient mice have less apoptotic cell death and lower caspase-3 activation related to decreased Bax levels.

With the expansion in male reproductive disorders, there is a pressing need to promote male fertility with natural products that are less likely to have negative effects, being more affordable and more compliant. Taurine is an essential β-amino acid that is abundant in most cells, especially excitable tissues (Schaffer and Kim 2018). Taurine has numerous physiological effects, including bile salt production in the liver, membrane stabilization, osmoregulation, neurotransmission modulation, Ca^2+^ homeostasis (Bhat et al. 2020), and mitigation of ER stress through improving protein folding (Ito et al. 2015). Taurine also has antioxidant and energy-producing properties, which help to protect cells (Schaffer et al. 2014; Shetewy et al. 2016). Taurine deficiency is often associated with serious diseases (Schaffer and Kim 2018).

Taurine provides a novel therapeutic option for metabolic illnesses such as diabetes mellitus (Ito et al. 2012), hypercholesterolemia (Sagara et al. 2015), and obesity (Yamori et al. 2010). Taurine has also been reported to improve many other diseases, including hypertension, atherosclerosis, ischemia–reperfusion injury, myocardial arrhythmias, congestive heart failure (Ahmadian et al. 2017; Qaradakhi et al. 2020), skeletal muscular dysfunction (Terrill et al. 2016), cerebral stroke, neurodegenerative diseases (Zhang et al. 2016), epilepsy (Oja and Saransaari 2013), and inflammatory diseases such as arthritis (Marcinkiewicz and Kontny 2014). In addition, taurine is extremely effective in the treatment of mitochondrial diseases such as mitochondrial encephalopathy (Rikimaru et al. 2012).

However, the roles of ER stress and mitochondrial injury in AlCl_3_-induced male gonadal damage have not been fully discussed, and the mechanisms of the taurine protective effect against AlCl_3_-induced male gonadal impairment remain to be studied in full. Therefore, the aim of this study is to investigate the role of ER stress and mitochondrial injury in the pathogenesis of AlCl_3_-induced testicular and epididymal damage at the histological, biochemical, and molecular levels, in addition to the possible ameliorative effect of taurine.

## Materials and methods

### Ethical approval statement

All the experimental work was performed following the international guidelines of Helsinki for animal welfare. The study was conducted in the Animal House, Faculty of Medicine, Alexandria University according to the guidelines for the care and use of experimental animals of Alexandria University, approved by the Institutional Animal Care and Use Committee with ethical committee approval IP (IRP no. 00012098-FWA NO:00018699).

### Chemicals

Aluminum chloride anhydrous powder (AlCl_3_, 99.999%, CAS no. 7446-70-0, EC no. 231-208-1, MDL no. MFCD00003422) was obtained from Sigma, Aldrich (St. Louis, MO, USA). To prepare stock solution (20 mg/ml), 2 g AlCl_3_ was dissolved in 100 ml distilled water. Taurine (Kosher, ≥ 98%) was obtained from Sigma Aldrich (Switzerland, CAS no. 107-35-7, EC no. 203-483-8, MDL no. MFCD 00,008,197) and dissolved in distilled water (62.5 mg/ml at 20 °C), water (65 mg/ml at 12 °C), and dimethylsulfoxide (DMSO) (< 1 mg/ml at 25 °C).

### Animals and study protocol

Forty-eight adult male albino Sprague–Dawley rats (90 days old, body weight 230 ± 30 g) were obtained from the Alexandria Experimental Research Center, Alexandria University. Rats were kept in specific pathogen-free conditions, fed normal animal chow, and exposed to a 12:12 h light/dark regime. Rats were housed in standard cages at a room temperature of 25 ± 2 °C and left to acclimatize for a few days before commencing the experimental procedures. Rats were indiscriminately and equally divided into four groups as follow:

Group I (negative control): rats orally received distilled water 5 ml/kg/day for 8 weeks. Group II (positive control): rats were intraperitoneally (IP) injected with taurine (200 mg/kg, once/day) (Gupte et al. 2019) for 8 weeks. Group III (AlCl_3_): Rats were orally administered AlCl_3_ (50 mg/kg/day) by gastric tube (Reichert et al. 2021) for 6 weeks. Group IV (AlCl_3_ plus taurine): Rats received IP taurine (200 mg/kg, once per day) for 2 weeks followed by a combination of oral AlCl_3_ (50 mg/kg/day) and IP taurine (200 mg/kg, once/day) for a further 6 weeks.

At the end of the experiment, animals were sacrificed under diethyl ether anesthesia. An abdominal incision was made, and testes and epididymis were carefully dissected. One testis and epididymis from each rat were kept in liquid nitrogen (at −80 °C) for further entire RNA derivation and quantitative real-time polymerase chain reaction (qRT-PCR) analysis of expression of *vimentin*, proliferating cell nuclear antigen (*PCNA*), *CHOP*, *Bcl-2*, *Bax*, and X-box DNA binding protein1 (*XBP1*) as well as estimation of malondialdehyde (MDA) and total antioxidant capacity (TAC). The other testis and epididymis were prepared for histological examination.

### Histological study

Specimens from the testis and epididymis were fixed in Bouin’s solution, dehydrated in an increasing series of alcohol, cleared in two changes of xylene, and then embedded in molten paraffin. Afterward, sections of 5–6 µm thickness were obtained using a rotary microtome, mounted on clean slides, and stained with hematoxylin and eosin (H&E) (Bancroft and Gamble 2008).

### Immunohistochemical (IHC) study of Bax and vimentin proteins

**Fig. 1:**
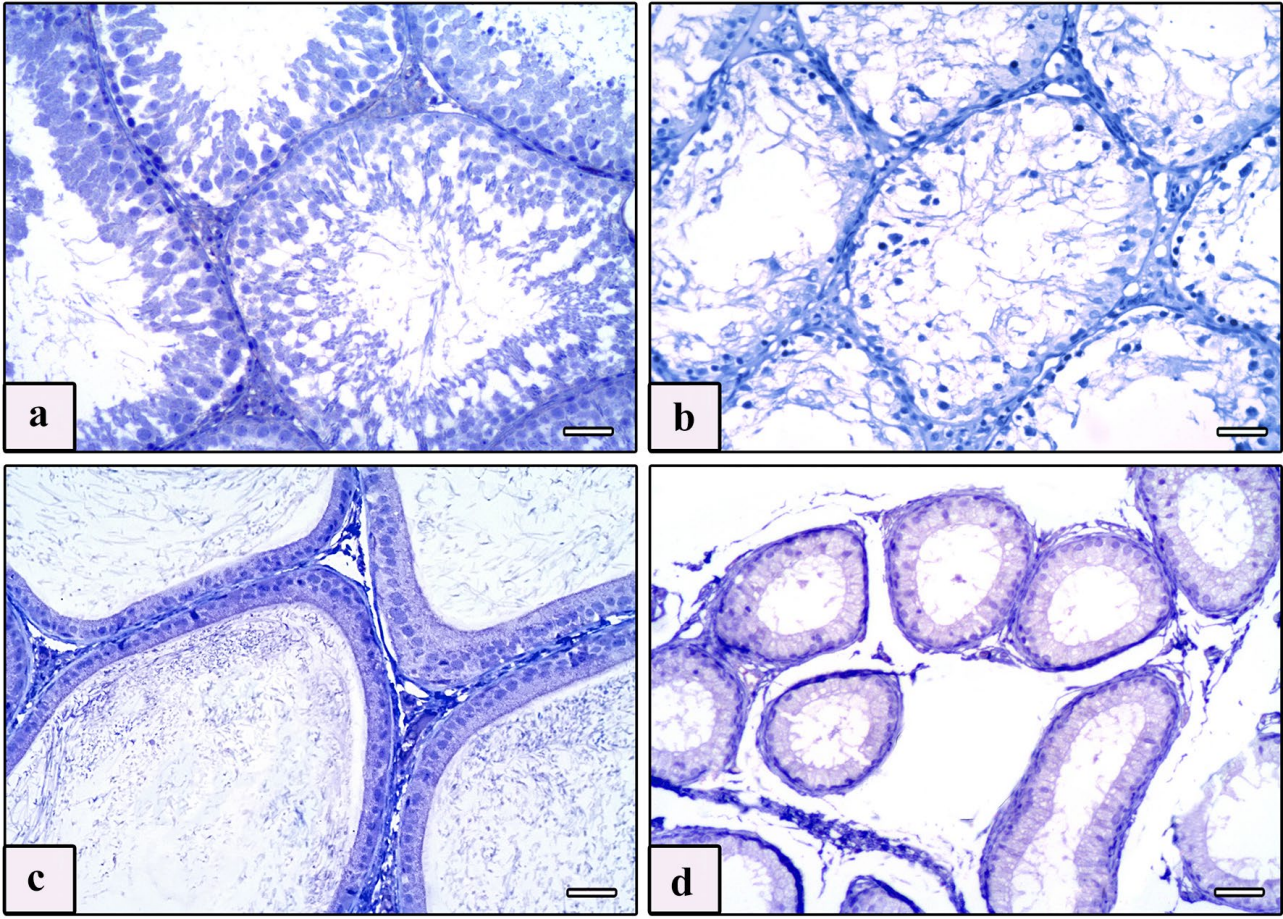
Photomicrographs of Bax and vimentin negative control sections of testis and epididymis of studied rat groups. **a** Negative control sections of the testis of the control groups. **b** Negative control sections of the testis of the AlCl_3_ group. **c** Negative control sections of the epididymis of the control groups. **d** Negative control sections of the epididymis of the AlCl_3_ group. Bax and Vimentin, scale bars = 50 μm (**a**–**d**)

Paraffin sections of testis and epididymis with thickness of 4 μm on positively charged glass slides were immunostained by the avidin–biotin procedure to detect Bax and vimentin proteins. The sections were deparaffinized in xylene and rehydrated in descending grades of alcohol down to distilled water, then treated with 0.3% hydrogen peroxide for 15 min to suppress endogenous peroxidase activity. For antigen retrieval, sections were boiled in citrate buffer (pH 6) for 10 min, then cooled and washed in phosphate-buffered saline (PBS). To diminish nonspecific reactions, sections were incubated with 10% normal goat serum for 30 min, followed by incubation for 1 h with antisera containing primary antibodies. Two primary antibodies were used. The first one was anti-Bax, rat monoclonal antibody (1:50, no. 13401A, clone G206-1276, immunoglobulin [Ig] M, 0.5 mg/mL, PharMingen, San Diego, CA) that was used in rats before according to Khalaf and El-Mansy (2019). The second one was anti-vimentin mouse monoclonal antibody (1:100, clone v9, Dako) that was used in rats before according to Ghoneim et al. (2014). After that, biotinylated secondary antibody (Dako-K0690; Dako Universal LSAB Kit) and streptavidin–biotin peroxidase complex (Dako-K0690) were added for 30 min. The immunoreactivity was visualized using 3,3′-diaminobenzidine (DAB)–hydrogen peroxide as a chromogen (Sigma-D5905; Sigma–Aldrich Company Ltd., Gillingham, UK) for 5 min. Mayer’s hematoxylin was used as counterstain. After replacing the primary antibodies with PBS, negative control sections were placed under the same conditions (Figs. 1a–d). The positive control slides for anti-Bax and anti-vimentin were rat thymus and tonsil, respectively (Cemek et al. 2008).

### Electron microscopic study

Small pieces (1 mm) of testis and epididymis were fixed in 2.5% glutaraldehyde buffered with 0.1 M sodium cacodylate (pH 7.2) for 2 h and washed three times with the same buffer. After washing, they were post-fixed in phosphate-buffered 1% osmium tetroxide for 2 h at room temperature, then dehydrated in an increasing series of ethanol. After immersion in propylene oxide, sections with thickness of 1 µm were obtained, stained with toluidine blue (1%), and inspected by light microscopy. Sections with thickness of 80–90 nm were cut by using a LKB ultramicrotome then stained with uranyl acetate and lead citrate (Woods and Stirling 2018). Ultrastructural details were inspected by transmission electron microscope (JEOL JEM-2100) (Gatan Inc., Tokyo, Japan) in the Electron Microscopic Unit, Faculty of Agriculture, Mansoura University, Egypt.

### Histomorphometrical study

#### Area percent of Bax and vimentin immunostaining

By image analysis, the area percent of Bax and vimentin immunostaining was measured by examining ten non-overlapping fields from each rat (five rats per group). The slides were photographed with an Olympus digital camera (E24-10 megapixel, China) equipped onto an Olympus microscope through a 0.5× photo-adaptor at magnification of 400×. The resulting images were analyzed on an Intel Core I3-based computer using Video Test Morphology software (Saint Petersburg, Russia) (Sabha et al. 2008).

#### Epididymal sperm count

The distal cauda of the epididymis was dissected, placed in separate microcentrifuge tubes, minced, and suspended in normal saline at 37 °C for 5 min. A light microscope with a plan objective lens was used to analyze the sperm count (Olympus CX31 Binocular Microscope. New York Microscope Company, NY, USA). The number of spermatozoa within ten grid squares was counted using a sperm counting chamber (Makler/Sperm Meter, Product Code: Sperm Counting Chamber; SEFI-Medical Instruments, New York, NY, USA). The chamber was prewarmed to 37 °C. Sperm count was expressed as million/ml (Karna et al. 2019a, b).

### Leydig cell count

Leydig cells were counted by examining ten non-overlapping sections from each group by an Olympus light microscope with a 0.5× photo-adaptor (OlympusBX40 image analyzer computer system, Cambridge, UK) using a calibrated ocular lens of a micrometer (Germany) to calculate the number per 100 μm^2^ (40× objective lens) (Moustafa et al. 2012; Alasmari et al. 2018).

### Biochemical and molecular studies

#### Measurement of MDA and TAC levels in testicular and epididymal tissues

The testicular and epididymal tissues were perfused with PBS solution, pH 7.4, containing 0.16 mg/ml heparin to remove red blood cells and clots. Then, each tissue sample was homogenized in 5–10 ml cold buffer (50 mM potassium phosphate, pH 7.5, 1 mM EDTA). Homogenates were centrifuged at 10,000 × *g* for 15 min at 4 °C. The supernatant fluid was separated into aliquots and stored at −80 °C until subsequent assessment of MDA and TAC.

MDA content was measured (in nmol/g tissue) by using the thiobarbituric acid (TBA) reaction as described previously by Draper et al. (1993). The absorbance of samples was measured at 532 nm using a colorimetric kit (cat. no. MD 2528). TAC was estimated (in ng/mg protein) by using commercially available colorimetric kits (Young 2001) (cat. no. # TA 25 12). Both kits were supplied by Bio-Diagnostics, Dokki, Giza, Egypt.

#### qRT-PCR of *vimentin*, *Bcl-2*, *Bax*, *PCNA*, *XBP1*, and *CHOP* gene expression in testicular and epididymal tissues

The total RNA was sequestrated from 25–35 mg tissue samples of rat testes and epididymis after snap freezing with liquid nitrogen using Tri-Fast reagent (PeqLab Biotechnologie GmbH, Carl-Thiersch St. 2B 91,052 Erlangen, Germany, cat. no. # 30–2010), triazole, and chloroform according to manufacturer’s instructions. Extracted RNA concentration and purity were assessed by Nanodrop 2000 spectrophotometer (Thermo Scientific, USA). Reverse transcription reaction for complementary DNA (cDNA) synthesis was accomplished with ~250 ng total RNA using the Maxima First Strand cDNA synthesis kit (Thermo Scientific, Waltham, MA, USA, cat no. #K1641). Testis and epididymis messenger RNA (mRNA) expression of *vimentin*, *Bcl-2*, *Bax*, *PCNA*, *XBP1*, and *CHOP* were measured by qRT-PCR using the Applied Biosystem 7500 RT-PCR detection system (Life Technology, USA) with HERAPLUS SYBR^®^ Green qPCR Master Mix (2x) (Willowfort, UK, cat. no. WF10308001). The RT-PCR reaction consisted of enzyme activation at 95 °C for 10 min, followed by 40 cycles of two-step cycling, including template denaturation at 95 °C (15 s), then annealing/extension at 60 °C (1 min). The primer sequences utilized were as follow: *vimentin*: forward, 5′-GCA CCC TGC AGT CAT TCA GA-3′; reverse, 5′- GCA AGG ATT CCA CTT TAC GTT CA-3′ (Zhuang et al. 2015); *Bcl-2*: forward, 5′-TGT GGA TGA CTG ACT ACC TGA ACC-3′; reverse, 5′-CAG CCA GGA GAA ATC AAA CAG AGG-3′ (Ghoneim et al. 2020); *Bax*: forward, 5′-GGC GAA TTG GCG ATG AAC TG-3′; reverse, 5′-ATG GTT CTG ATC AGC TCG GG-3′ (AL-Megrin et al. 2020); *PCNA*: forward, 5′- CTG CTG GGA CAT CAG TTC GG-3′; reverse, 5′-GAT CGC AGC GGT ATG TGT CG-3′ (Duan et al. 2020); *XBP1*: forward, 5′- GGT ATA GCC AGC GAG TGC T-3′; reverse, 5′-TCA GTT GGG AGC CTG ATT CT-3′ (Zhu et al. 2012); *CHOP*: forward, 5′-GAAAGCAGAAACCGGTCCAAT-3′; reverse, 5′-GGATGAGATATAGGTGCCCCC-3′ (Idari et al. 2021); *β-actin* (internal control gene): forward, 5′-AAG ATC CTG ACC GAG CGT GG-3′; reverse, 5′-CAG CAC TGT GTT GGC ATA GAG G-3′ (Ghoneim et al. 2020). The expression of the analyzed genes was normalized to that of the internal control gene β-actin using the comparative ∆∆CT method.

### Statistical analysis

The obtained data were managed, and analyzed using the Statistical Package for Social Sciences (SPSS) version 15 for Windows (SPSS Inc, Chicago, IL, USA). Quantitative data were first verified for normality by the Kolmogrov–Smirnov test. As the presented data showed a parametric distribution, they are presented as mean ± standard deviation (SD). The *F* test (one-way analysis of variance test) was applied to compare between more than two groups, followed by Tukey’s post hoc test. Differences were considered statistically significant for *P* value < 0.05 (Landau and Everitt 2003).

## Results

### Light microscopic results

#### H&E stain

**Fig. 2:**
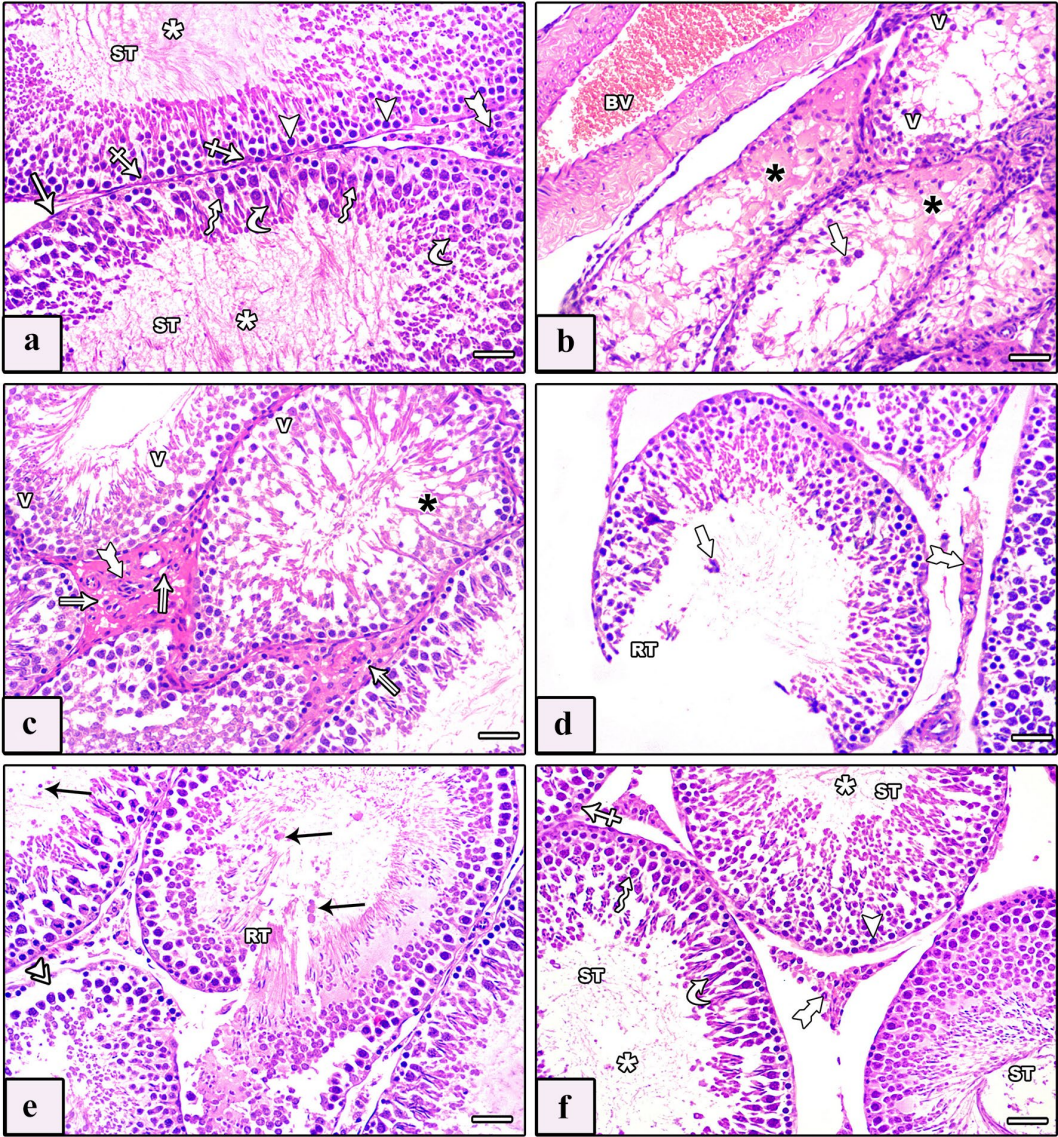
Photomicrographs of sections of testis of studied rat groups stained with H&E. **a** Control group shows thin regular basement membrane ensheathed by flattened nuclei of myoid cells (arrow). The seminiferous tubule (ST) is lined by spermatogenic cells and Sertoli cells (crossed arrows). Spermatogenic cells include spermatogonia (arrowheads), primary spermatocytes (zigzag arrows) and spermatids (curved arrows) with intraluminal spermatozoa (white asterisk). The tubules are separated by interstitial tissue showing Leydig cells (tailed arrow). **b**–**e** AlCl_3_ group shows a disorganized testicular architecture. **b** Congested thickened dilated blood vessel (BV) is seen. Seminiferous tubules exhibit degeneration and irregularity with loss of their lining (black asterisk), multiple vacuoles (V) between spermatogenic cells, and large multinucleated cells (thick arrow) within the lumen of some tubules. **c** Different shapes and wavy outlines of seminiferous tubules with loss of their epithelial lining (black asterisk) and multiple vacuoles (V) between them are seen. Degenerated Leydig cells with darkly stained nuclei and vacuolated (double arrow) or acidophilic (tailed arrow) cytoplasm are seen. **d** Ruptured tubules (RT) with large multinucleated cells (thick arrow) within their lumen and wide interstitial space containing degenerated Leydig cells with acidophilic cytoplasm (tailed arrow) are seen. **e** Ruptured tubules (RT) are seen with exfoliated pyknotic cells in the lumen (arrows), others show degenerated detached spermatogenic cells from the basement membrane (double arrowhead). **f** AlCl_3_ plus taurine group shows normal lining of seminiferous tubules (ST), spermatogonia (arrowhead), primary spermatocyte (zigzag arrow), spermatid (curved arrow), and Sertoli cells (crossed arrow). Note the presence of spermatozoa in their lumens (white asterisk) and less wide interstitial tissue containing normal Leydig cells (tailed arrow). H&E, scale bars = 50 μm (**a**–**f**)

Inspection of testicular tissues of control groups revealed the standard construction of testis structures. Seminiferous tubules (STs) were bounded by a basement membrane with myoid cells. They were lined by stratified spermatogenic cells in addition to Sertoli cells. The spermatogenic cells included spermatogonia, primary spermatocytes, spermatids (rounded and elongated), and spermatozoa. Between STs, the interstitium contained Leydig cells and blood vessels. Leydig cells appeared in clusters or singly, having rounded vesicular nuclei with extensive acidophilic cytoplasm and variable numbers of lipid vacuoles (Fig. 2a).

The AlCl_3_ group showed apparent distortion in the testicular architecture. STs exhibited many disturbances in the form of degeneration, irregularity, different shapes with wavy outlines, and loss of the normal distribution of the epithelial lining, with the presence of multiple vacuoles between them (Fig. 2b, c). Congested thickened dilated blood vessels, and degenerated spermatogenic cells with multiple large multinucleated cells were noticed (Fig. 2b). Degenerated Leydig cells with darkly stained nuclei and vacuolated or acidophilic cytoplasm were apparent (Fig. 2c). There was a rupture of some tubules. Large multinucleated cells were seen within the lumen of some ruptured tubules with wide interstitial space comprising degenerated Leydig cells with acidophilic cytoplasm (Fig. 2d). Some ruptured tubules exhibited exfoliated pyknotic cells in the lumen, and others showed degenerated spermatogenic cells detached from the basement membrane (Fig. 2e).

Examination of the AlCl_3_ plus taurine group revealed preserved testicular structure that was almost similar to control groups. The STs were lined by stratified spermatogenic epithelium based on the basement membrane. It contained spermatozoa in the lumen and was disconnected by a less wide interstitial tissue containing Leydig cells. Leydig cells appeared with rounded vesicular nuclei and extensive acidophilic cytoplasm (Fig. 2f).

**Fig. 3:**
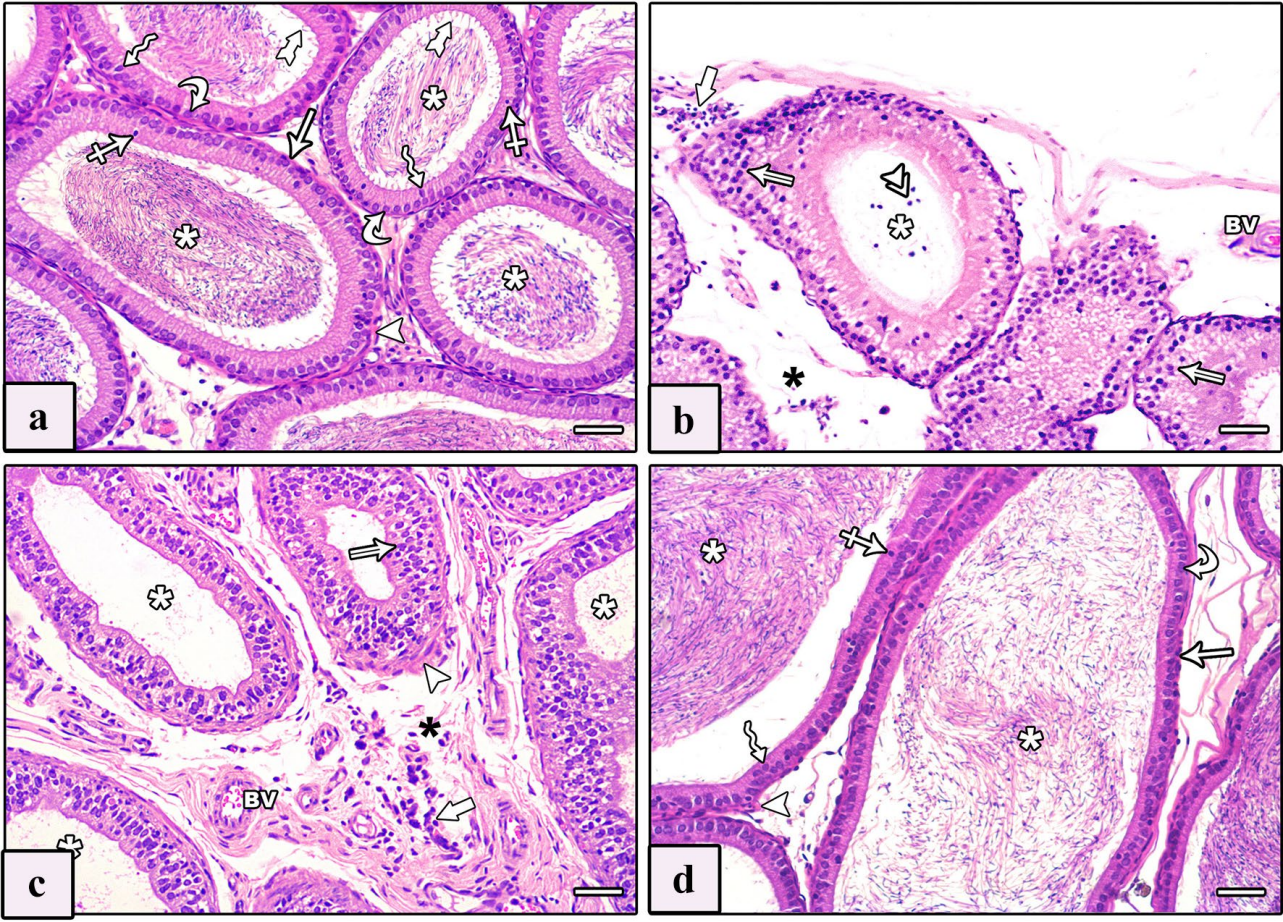
Photomicrographs of sections of epididymis of studied rat groups stained with H&E. **a** Control group: the epididymis exhibits regular spherical tubules lined by pseudostratified columnar epithelium with stereocilia (tailed arrows) based on a thin basement membrane (arrow) and surrounded by thin layers of circular smooth muscle (arrowhead). The epithelium shows three types of cells: principal (zigzag arrows), basal (curved arrow), and apical (crossed arrows). A plug of sperm is seen in the tubular lumen (asterisk). **b**, **c** AlCl_3_ group: epididymal lining is irregular, stratified with vacuolated cytoplasm (double arrows) and surrounded by a thick layer of smooth muscle fibers (arrowhead). There are many exfoliated cells inside the lumen (double arrowhead), reduced spermatozoa in the lumen of some tubules with the presence of empty ones (white asterisk), and wide interstitium (black asterisk) containing congested dilated blood vessel (BV) and mononuclear cellular infiltration (thick arrow). **d** AlCl_3_ plus taurine group: the epididymis shows normal epithelial lining of tubules, with principal (zigzag arrows), basal (curved arrow), and apical (crossed arrows) cells based on a thin basement membrane (arrow) surrounded by thin layers of circular smooth muscle (arrowhead). A plug of sperm (white asterisk) is seen in the lumen of tubules. H&E, scale bars = 50 μm (**a**–**d**)

The epididymis of the control groups exhibited regular spherical tubules lined by pseudostratified columnar epithelium with stereocilia based on a thin basement membrane that was surrounded by thin layers of circular smooth muscle. The epithelium showed three types of cells: principal, basal, and apical. A plug of sperm was seen in the tubular lumen. The extratubular interstitium contained blood vessels (Fig. 3a). Epididymis of the AlCl_3_ group revealed fewer spermatozoa in the lumen of some tubules, while others were empty. Its lining was irregular, stratified, and surrounded by a thick layer of smooth muscle fibers with vacuolated cytoplasm and many exfoliated cells inside the lumen. Wide interstitium contained congested dilated blood vessels, and mononuclear cellular infiltration was seen (Figs. 3b , c). In the AlCl_3_ plus taurine group, the epididymis was almost identical to that of the control group, exhibiting regular spherical tubules lined by pseudostratified columnar ciliated epithelium based on a thin basement membrane encircled by thin layers of circular smooth muscles (Fig. 3d).

### Immunohistochemical stains

#### Anti-Bax

**Fig. 4:**
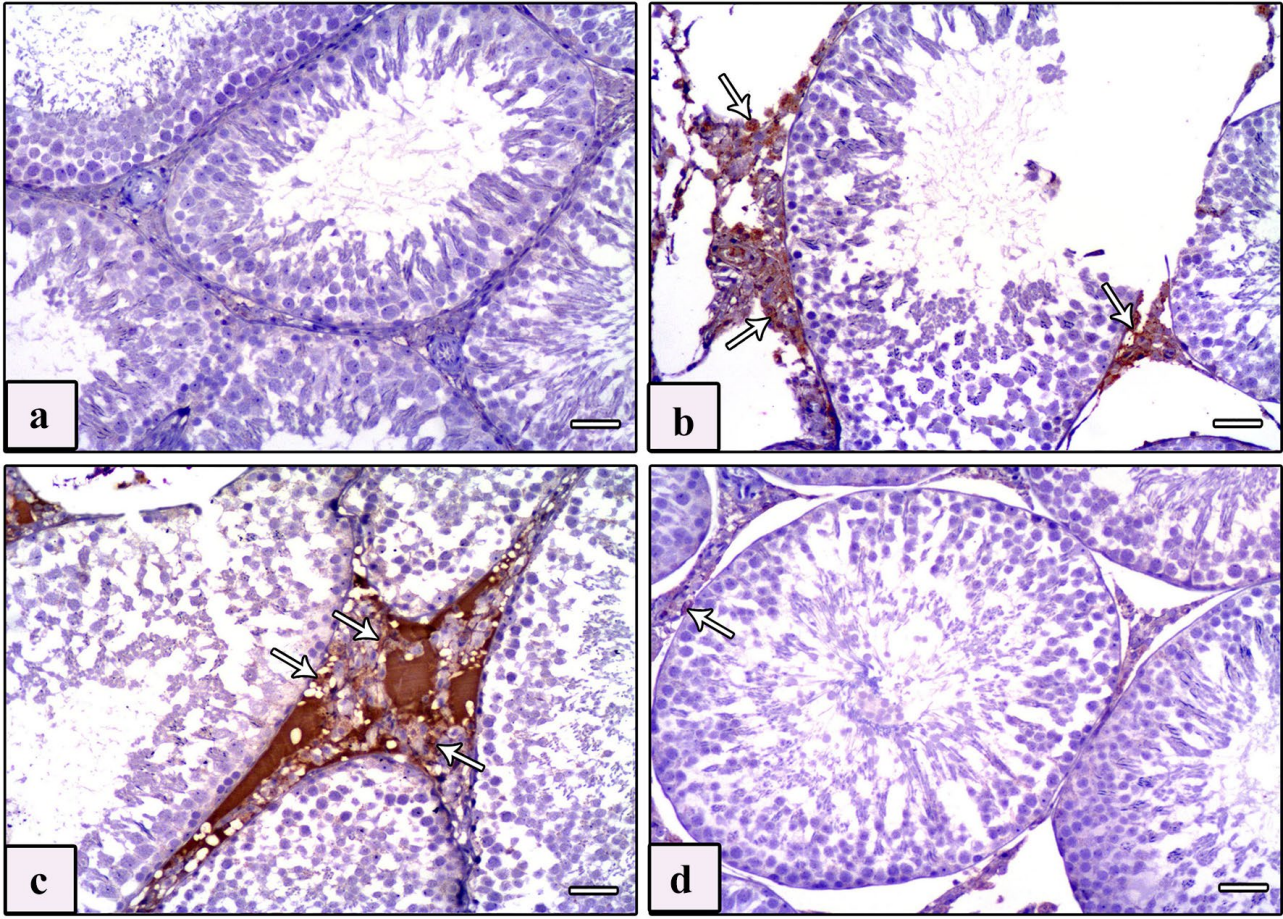
Photomicrographs of sections of testis of studied rat groups stained with Bax immunostaining. **a** Control group shows negative immunoreaction in the cytoplasm of the interstitial cell of Leydig. **b**, **c** AlCl_3_ group reveals intensive immunoreaction in the cytoplasm of interstitial cells of Leydig (arrows). **d** AlCl_3_ plus taurine group shows minimal immunoreaction in the cytoplasm of interstitial cells of Leydig (arrows). Bax, scale bars = 50 μm (**a**–**d**)

**Fig. 5:**
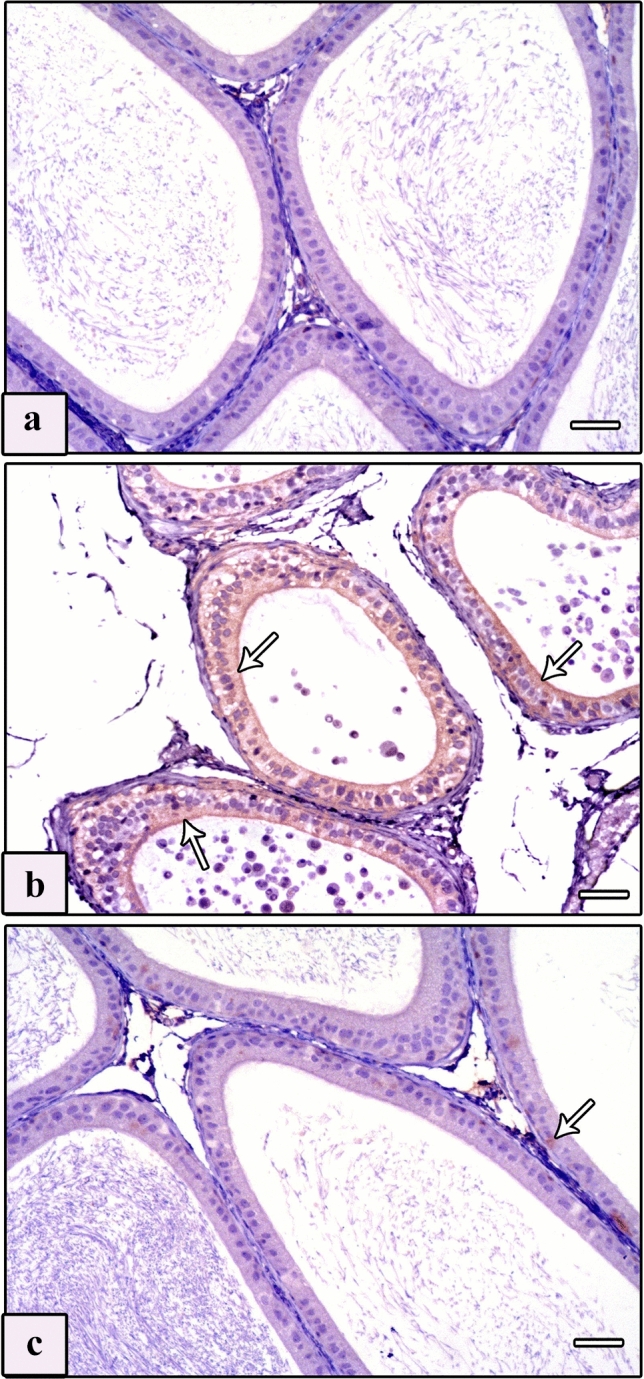
Photomicrographs of sections of epididymis of studied rat groups with Bax immunostaining. **a** Control group shows negative immunoreaction in the epididymal cell lining. **b** AlCl_3_ group reveals intensive immunoreaction in approximately all epididymal cell lining (arrows). **c** AlCl_3_ plus taurine group shows minimal immunoreaction in the epididymal cell lining (arrows). Bax, scale bars = 50 μm (**a**–**c**)

Examination of Bax immunostained sections of the testis of control groups displayed negative cytoplasmic immunoreaction in the interstitial cells of Leydig (Fig. 4a), whereas immunostained sections from the AlCl_3_ group showed intensive immunoreaction (Fig. 4b, c). In the immunostained sections of the AlCl_3_ plus taurine group, the immunoreaction in the cytoplasm of the interstitial cells of Leydig was negligible (Fig. 4d). Examination of Bax immunostained sections of the epididymis revealed negative immunoreaction in the epididymal cell lining of control groups (Fig. 5a). Intensive immunoreaction in almost the whole epididymal cell lining of the AlCl_3_ group was noticed (Fig. 5b). In the AlCl_3_ plus taurine group, the immunoreaction in the epididymal cell lining was minimal (Fig. 5c).

#### Anti-vimentin

**Fig. 6:**
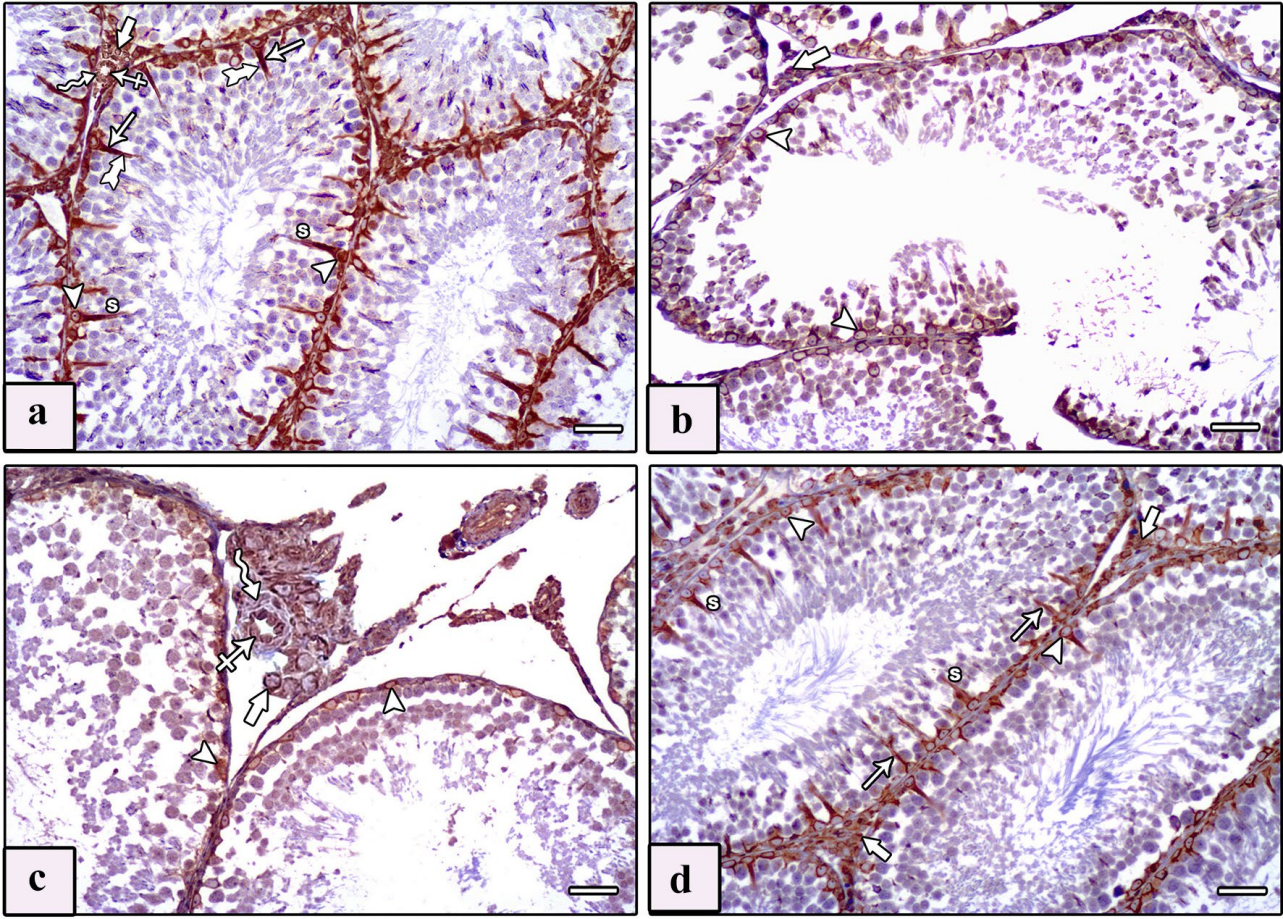
Photomicrographs of sections of testis of studied rat groups stained with vimentin immunostaining. **a** Control group shows a strong brown, positive immune reaction in the cytoplasm of Sertoli cells (S) with characteristic flame-like vimentin immune reaction located in the perinuclear regions (arrowhead), midportions (arrow), and apices (tailed arrow) of their cytoplasm. Intensive positive immune reaction is seen in the cytoplasm of Leydig cells (thick arrow), the cytoplasm of endothelial cells of the blood vessel (crossed arrow), and in the smooth muscle cells (zigzag arrow). **b**, **c** Vimentin-immunostained sections of testes of AlCl_3_ group reveal a positive immunoreaction limited to the perinuclear region of Sertoli cells (arrowhead) with loss of the typical flame-like appearance as well as a positive immune reaction in the interstitial Leydig cells (thick arrow), a moderate reaction in the endothelial cells of blood vessels (crossed arrow), and a faint reaction in smooth muscle layer (zigzag arrow). **d** Vimentin-immunostained sections of testes of AlCl_3_ plus taurine group reveal a flame-like positive cytoplasmic immunoreaction spreading from the perinuclear region (arrowhead) to the apical parts (arrows) of the majority of Sertoli cells (S). Also, a positive immune reaction is seen in Leydig cells (thick arrows). Vimentin, scale bars = 50 μm (**a**–**d**)

**Fig. 7:**
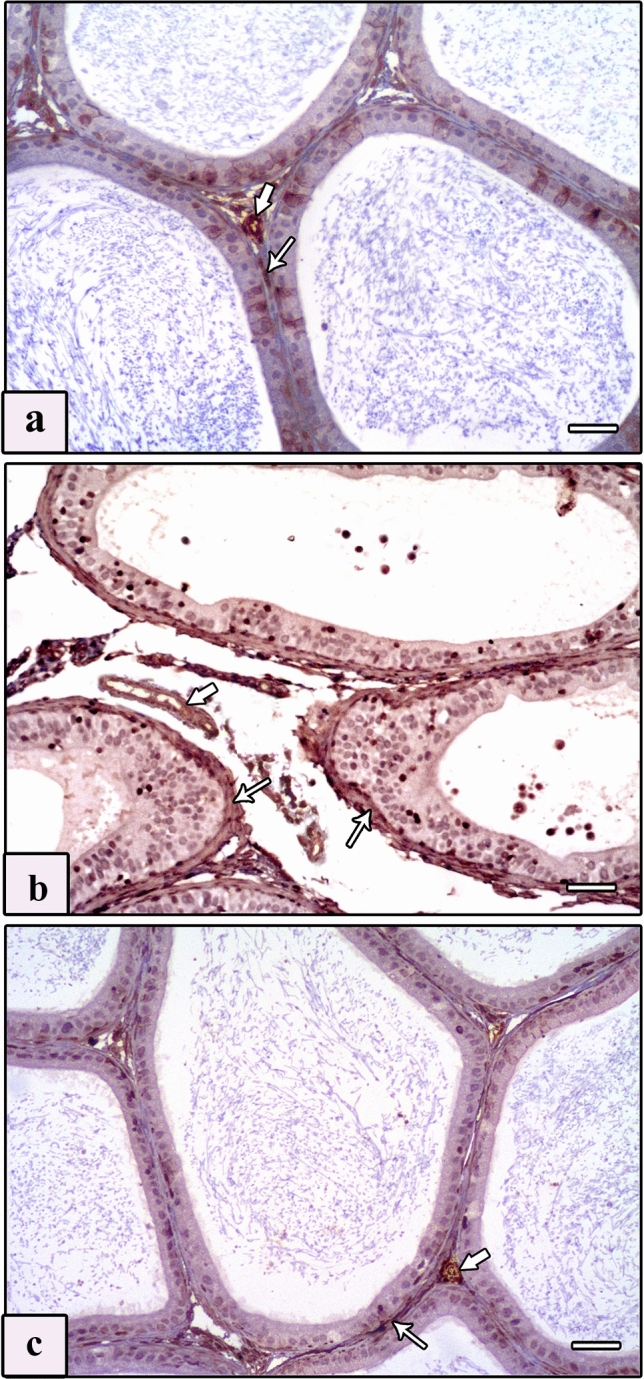
Photomicrographs of sections of epididymis of studied rat groups stained with vimentin immunostaining. **a** Control group shows a strong immune reaction in the peritubular myoid cells (arrow), smooth muscle layer (curved arrow), and cytoplasm of endothelial cells of blood vessels (thick arrow) present in the interstitial tissue between the epididymal ducts. **b** AlCl_3_ group shows a faint immune reaction in the peritubular myoid cells (arrow), thickened smooth muscle layer (curved arrow), and cytoplasm of endothelial cells (thick arrow) **c** AlCl_3_ plus taurine group reveals a strong immune reaction in the peritubular myoid cells (arrow), smooth muscle layer (curved arrow), and cytoplasm of endothelial cells (thick arrow). Vimentin, scale bars = 50 μm (**a**–**c**)

Examination of vimentin-immunostained sections of testis of control groups revealed typical flame-like vimentin immunoreaction of Sertoli cells. The reaction was located in the perinuclear regions and midportions, and increasingly reached the apex of the cytoplasm. A robust positive immune reaction was detected in the cytoplasm of Leydig cells, endothelial cells of blood vessels, and smooth muscle cells (Fig. 6a). However, vimentin-immunostained sections of testes of the AlCl_3_ group revealed a positive immunoreaction limited to the perinuclear region of Sertoli cells with loss of the typical flame-like appearance as well as a positive immunoreaction in the interstitial Leydig cells and a moderate reaction in the blood vessels (Fig. 6b, c). Meanwhile, vimentin-immunostained sections of testes of the AlCl_3_ plus taurine group revealed a flame-like positive cytoplasmic immunoreaction spreading to the apical parts of the majority of Sertoli cells. Moreover, a positive immune reaction was detected in the Leydig cells and blood vessels (Fig. 6d). Examination of vimentin-immunostained sections of the epididymis of control groups revealed strong immune reaction in the peritubular myoid cells, the smooth muscle layer, and the cytoplasm of endothelial cells of blood vessels in the interstitial tissue between the epididymal ducts (Fig. 7a). However, vimentin-immunostained sections of the epididymis of the AlCl_3_ group revealed a faint immune reaction in the peritubular myoid cells, thickened smooth muscle layer, and cytoplasm of endothelial cells (Fig. 7b). Vimentin-immunostained sections of the epididymis of the AlCl_3_ plus taurine group exhibited restoration of the standard immune reaction as in control groups (Fig. 7c).

#### Electron microscopic results

**Fig. 8:**
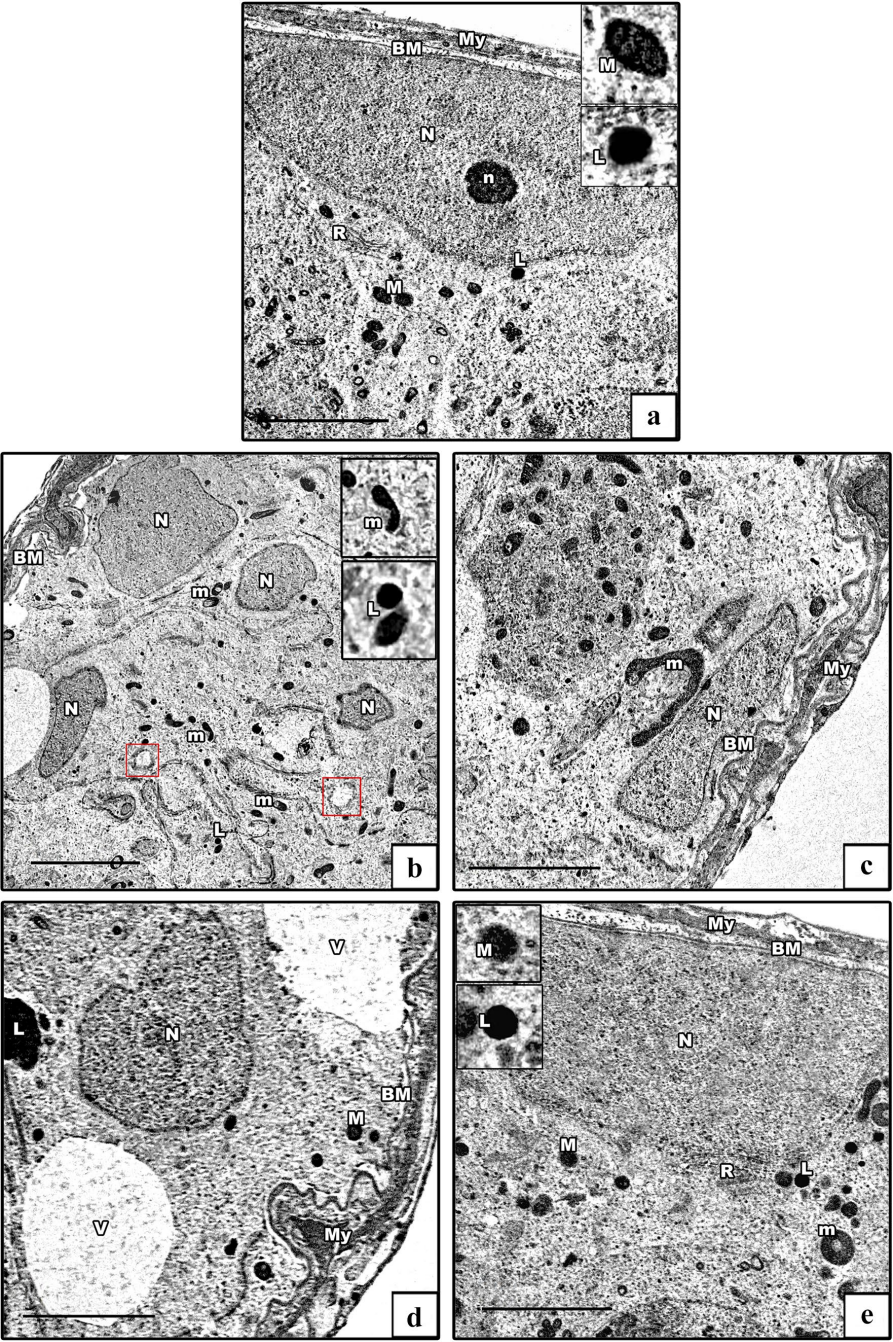
Electron micrographs of Sertoli cells. **a** Control group shows Sertoli cell resting on a regular basement membrane (BM) with myoid cell (My). It has a large oval euchromatic nucleus (N) with a prominent nucleolus (n). Its cytoplasm shows mitochondria (M), rER (R), and lysosomes (L). The insets show normal mitochondria with preserved cristae and lysosomes. **b**–**d** AlCl_3_ group shows distorted Sertoli cells resting on thickened irregular basement membrane (BM) containing myoid cells (My). They have irregular heterochromatic nuclei (N), ring-shaped and vacuolated mitochondria (m), swollen rER (red box), lysosomes (L), and vacuolated cytoplasm (V). The insets in b show degenerated ring-shaped mitochondria and lysosomes. **e** AlCl_3_ plus taurine group: normal Sertoli cell resting on a thin regular basement membrane (BM) with myoid cell (My). It has an oval nucleus (N), normal mitochondrion (M), normal rER (R), and lysosomes (L). The insets show normal mitochondria with preserved cristae and lysosomes. Notice the presence of some vacuolated mitochondria. Transmission electron microscope, scale bars = 5 μm (**a**, **c**, **d**, **e**), 10 μm (**b**)

Electron microscopic (EM) investigation of the ultrathin sections of the testes of control groups showed Sertoli cells lying on a regular thin basement membrane with myoid cells. They were huge oval cells that had large indented euchromatic nucleus with a prominent nucleolus. Their cytoplasm exhibited mitochondria, rough ER (rER), and ribosomes, besides lysosomes (Fig. 8a). The AlCl_3_ group showed an apparent increase in the number of distorted Sertoli cells resting on thickened irregular basement membrane with myoid cells. Irregular heterochromatic nuclei of Sertoli cells with ring-shaped and vacuolated mitochondria, swollen rER, many lysosomes, and cytoplasmic vacuolations were seen (Fig. 8b–d). The AlCl_3_ plus taurine group displayed Sertoli cell resting on normal basement membrane with maintained ultrastructural picture despite the presence of some vacuolated mitochondria (Fig. 8e).

**Fig. 9:**
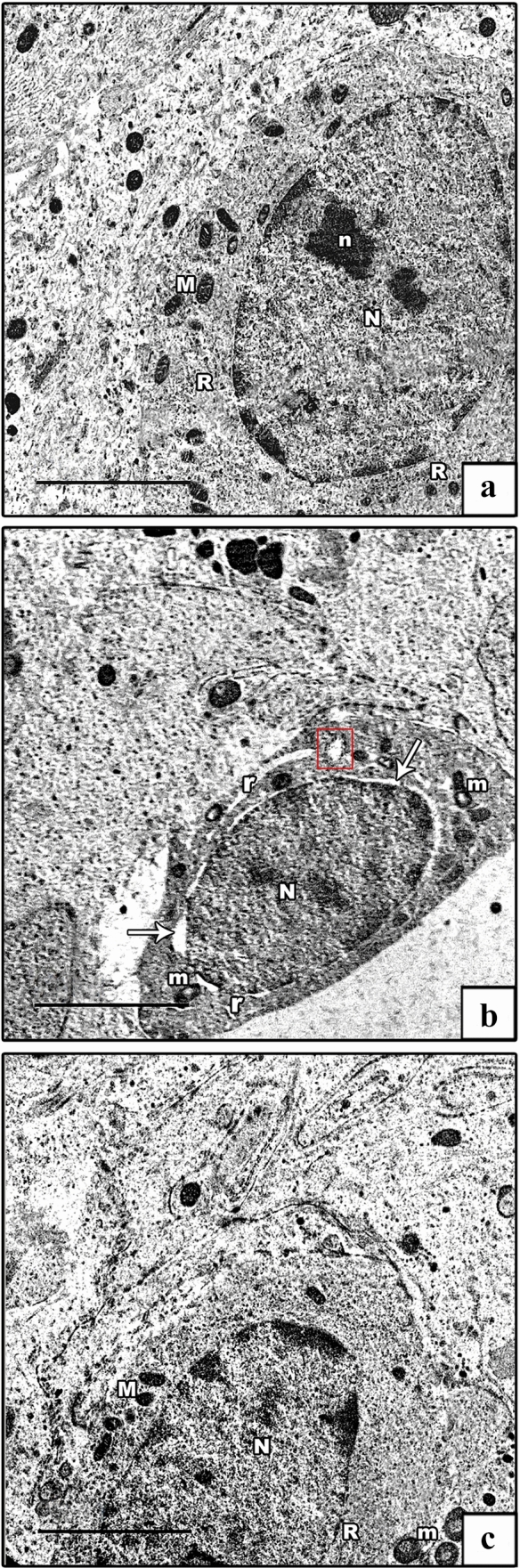
Electron micrographs of spermatogonia. **a** Control group shows a spermatogenic cell with an oval euchromatic nucleus (N) with a prominent nucleolus (n). Its cytoplasm shows mitochondria (M) with characteristic cristae and rER (R). **b** AlCl_3_ group shows distorted spermatogenic cell. It has a heterochromatic nucleus (N) with a wide perinuclear space (arrow), vacuolated mitochondrion (m), dilated rER (r), and swollen rER (red box). **c** AlCl_3_ plus taurine group: normal spermatogenic cell with an oval euchromatic nucleus (N), normal mitochondria (M), and normal rER (R). Note the presence of some vacuolated mitochondria (m). Transmission electron microscope, scale bars = 5 μm (**a**–**c**)

Spermatogonia of the control groups exhibited oval euchromatic nuclei with prominent nucleolus and peripheral heterochromatin clumps, and its cytoplasm contained mitochondria with characteristic cristae (Fig. 9a). The AlCl_3_ group showed distorted spermatogonia with heterochromatic nuclei and dilated perinuclear spaces. Their cytoplasm showed degenerated mitochondria and dilated swollen rER (Fig. 9b). The AlCl_3_ plus taurine group displayed spermatogonia with preserved ultrastructural picture apart from the presence of some vacuolated mitochondria (Fig. 9c).

**Fig. 10:**
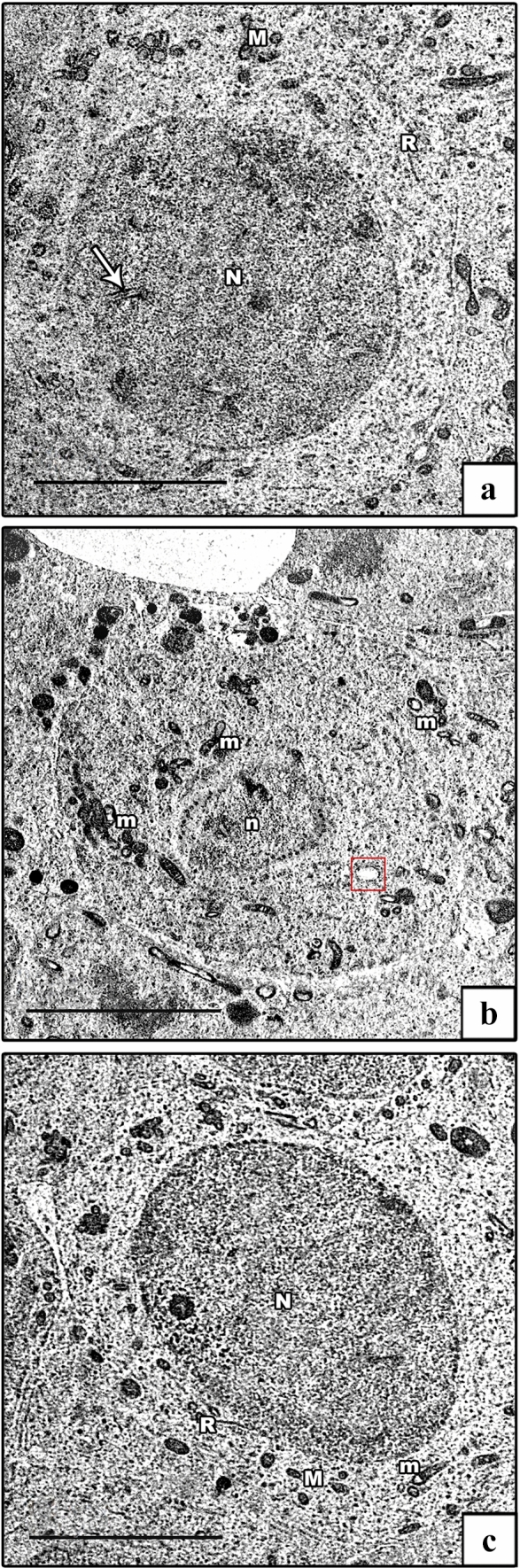
Electron micrographs of primary spermatocyte. **a** Control group demonstrates a primary spermatocyte with an oval euchromatic nucleus (N) showing synaptonemal complex (arrow). Its cytoplasm shows rER (R) and peripherally arranged mitochondria (M). **b** AlCl_3_ group shows distorted primary spermatocyte. It has a heterochromatic shrunken nucleus (n), swollen rER (red box), and vacuolated mitochondria (m). **c** AlCl_3_ plus taurine group: normal primary spermatocyte with an oval euchromatic nucleus (N), normal rER (R), normal mitochondria (M), and vacuolated one (m). Transmission electron microscope, scale bars = 5 μm (**a**–**c**)

The ultrastructure of primary spermatocytes of the control groups showed cells with rounded euchromatic nuclei displaying synaptonemal complexes. Their cytoplasm revealed peripherally arranged mitochondria (Fig. 10a). The AlCl_3_ group showed primary spermatocytes with shrunken heterochromatic nuclei. Their cytoplasm revealed vacuolated mitochondria besides dilated and swollen rER (Fig. 10b). The AlCl_3_ plus taurine group demonstrated conservation of the normal ultrastructural appearance of primary spermatocytes despite the presence of some vacuolated mitochondria (Fig. 10c).

**Fig. 11:**
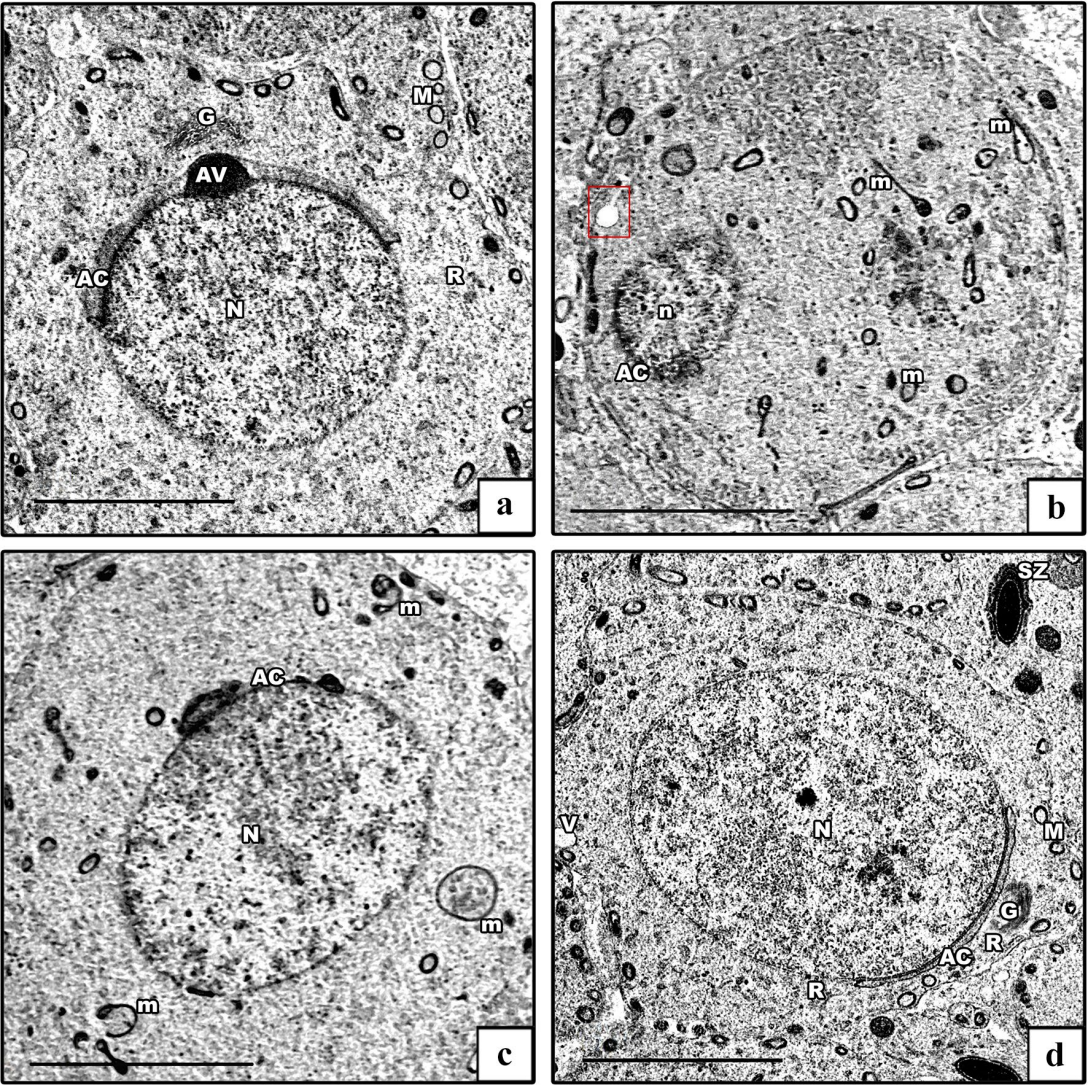
Electron micrographs of rounded spermatid. **a** Control group demonstrates a rounded spermatid with a spherical euchromatic nucleus (N) with a prominent acrosomal cap (AC) and an acrosomal vesicle (AV). Its cytoplasm shows a well-developed Golgi apparatus (G), rER (R), and mitochondria (M). **b**, **c** AlCl_3_ group. **b** Degenerated rounded spermatid with a shrunken heterochromatic nucleus (n) and abnormal acrosomal cap (AC) are seen. Its cytoplasm shows a swollen rER (red box) and vacuolated mitochondria (m). **c** Early spermatid with an euchromatic nucleus (N) and ill-developed acrosomal cap (AC) are seen. Its cytoplasm shows vacuolated and ring-shaped mitochondria (m). **d** AlCl_3_ plus taurine group: normally rounded spermatid with a spherical euchromatic nucleus (N) with prominent acrosomal cap (AC). The cytoplasm shows a well-developed Golgi apparatus (G), normal rER (R), mitochondria (M), and some vacuoles (V). Note the presence of spermatozoa (SZ). Transmission electron microscope, scale bars = 5 μm (**a**–**d**)

EM examination of ultrathin sections of the early spermatids of the control groups showed rounded spermatids with spherical euchromatic nuclei. They had regular acrosomal caps covering the anterior parts of the nuclei, linked with dense acrosomal granules. Their cytoplasm revealed a well-formed obvious Golgi and peripheral mitochondria (Fig. 11a). In the AlCl_3_ group, the spermatids had degenerated. Some cells had shrunken heterochromatic nuclei, and others showed euchromatic nuclei and ill-developed acrosomal caps. The cytoplasm showed degenerated, vacuolated, and ring-shaped mitochondria in addition to dilated and swollen rER (Figs. 11b, c). The AlCl_3_ plus taurine group demonstrated a comparatively normal ultrastructural picture despite the presence of vacuolated cytoplasm (Fig. 11d).

**Fig. 12:**
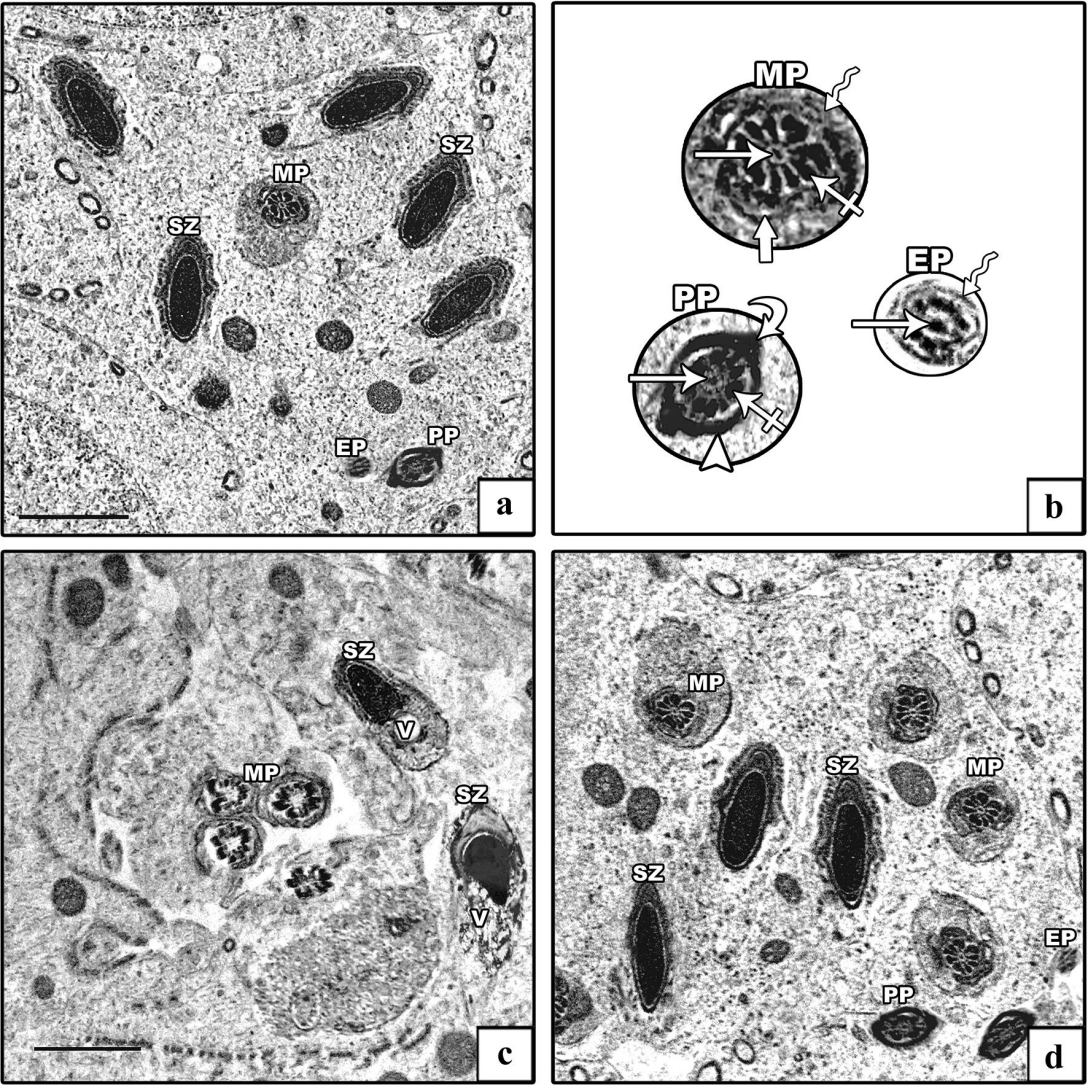
Electron micrographs of spermatozoa. **a**, **b** Control group shows the spermatozoa head with an elongated electron-dense nucleus (SZ). The tail has its middle piece (MP), principal piece (PP), and end piece (EP). The middle piece comprises (from inside to out) central axoneme (arrow), nine outer dense fibers (crossed arrow), mitochondrial sheath (thick arrow), and plasma membrane (zigzag arrow). The principal piece comprises a central axoneme (arrow), nine outer dense fibers (crossed arrow), and two longitudinal columns (curved arrow) connected by transverse circumferential ribs (arrowhead). The end piece comprises the central axoneme (arrow) and plasma membrane (zigzag arrow). **c** AlCl_3_ group: degenerated head of spermatozoa (SZ) with vacuolation in its retained cytoplasm (V) is seen with abnormal outlines and incomplete mitochondrial sheath of the middle piece (MP). **d** AlCl_3_ plus taurine group exhibits a normal picture of the spermatozoa head (SZ) as well a middle piece (MP), principal piece (PP), and end piece (EP) of the tail. Transmission electron microscope, scale bars = 2 μm (**a**, **c**, **d**)

The ultrastructure of spermatozoa of the control groups displayed their distinctive appearance; the heads exhibited an elongated electron-dense nuclei, while transverse sections of the tails exhibited middle, principal, and end pieces (Fig. 12a). Each middle piece comprised (from inside to out) central axoneme, nine outer dense fibers, mitochondrial sheath, and plasma membrane. Principal piece was composed of a central axoneme, nine dense outer fibers, two longitudinal columns (ventral and dorsal) connected by transverse circumferential ribs, and a plasma membrane. The end piece comprised a central axoneme and plasma membrane (Fig. 12b). The AlCl_3_ group showed spermatozoa having degenerated heads and many vacuolations in their retained cytoplasm. The middle pieces of their tails showed abnormal outlines with incomplete mitochondrial sheaths (Fig. 12c). The AlCl_3_ plus taurine group exhibited a normal ultrastructural picture of the spermatozoa regarding their heads and tails (Fig. 12d).

**Fig. 13:**
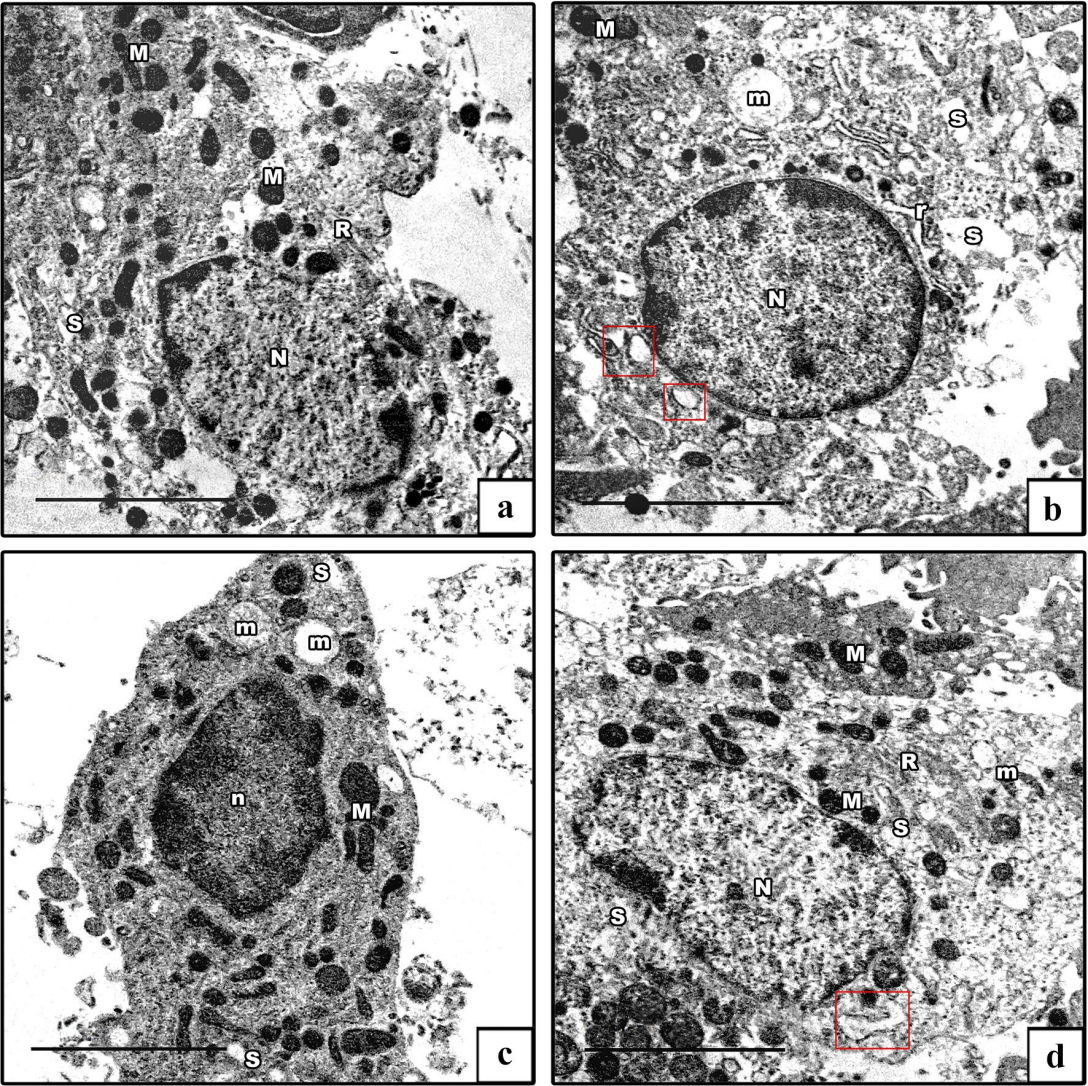
Electron micrographs of Leydig cells. **a** Control group demonstrates Leydig cells with an oval euchromatic nucleus (N), sER (S), rER (R), and mitochondria (M). **b**, **c** AlCl_3_ group: degenerated Leydig cells. Some cells have irregular nuclei (N), and others have shrunken heterochromatic nuclei (PN). The cytoplasm reveals dilated sER (s), dilated rER (r), swollen rER (red box), normal mitochondria (M), and vacuolated ones (m) **d** AlCl_3_ plus taurine group shows normal Leydig cells with normal mitochondria (M) and normal rER (R) apart from some dilated rER (red box) and some vacuolated mitochondria (m). Transmission electron microscope, scale bars = 5 μm (**a**–**d**)

The ultrastructure of Leydig cells of the control groups showed normal structural details with oval euchromatic nuclei, cytoplasm revealed mitochondria, smooth ER (sER), and rER (Fig. 13a). The AlCl_3_ group showed degenerated Leydig cells. Some cells appeared with euchromatic nuclei, and others with shrunken heterochromatic nuclei. The cytoplasm revealed dilated and swollen both sER and rER, besides degenerated and vacuolated mitochondria (Fig. 13b, c). The AlCl_3_ plus taurine group displayed Leydig cells with a well-preserved ultrastructural picture despite some vacuolated mitochondria and dilated sER in their cytoplasm (Fig. 13d).

#### Histomorphometrical results

**Fig. 14:**
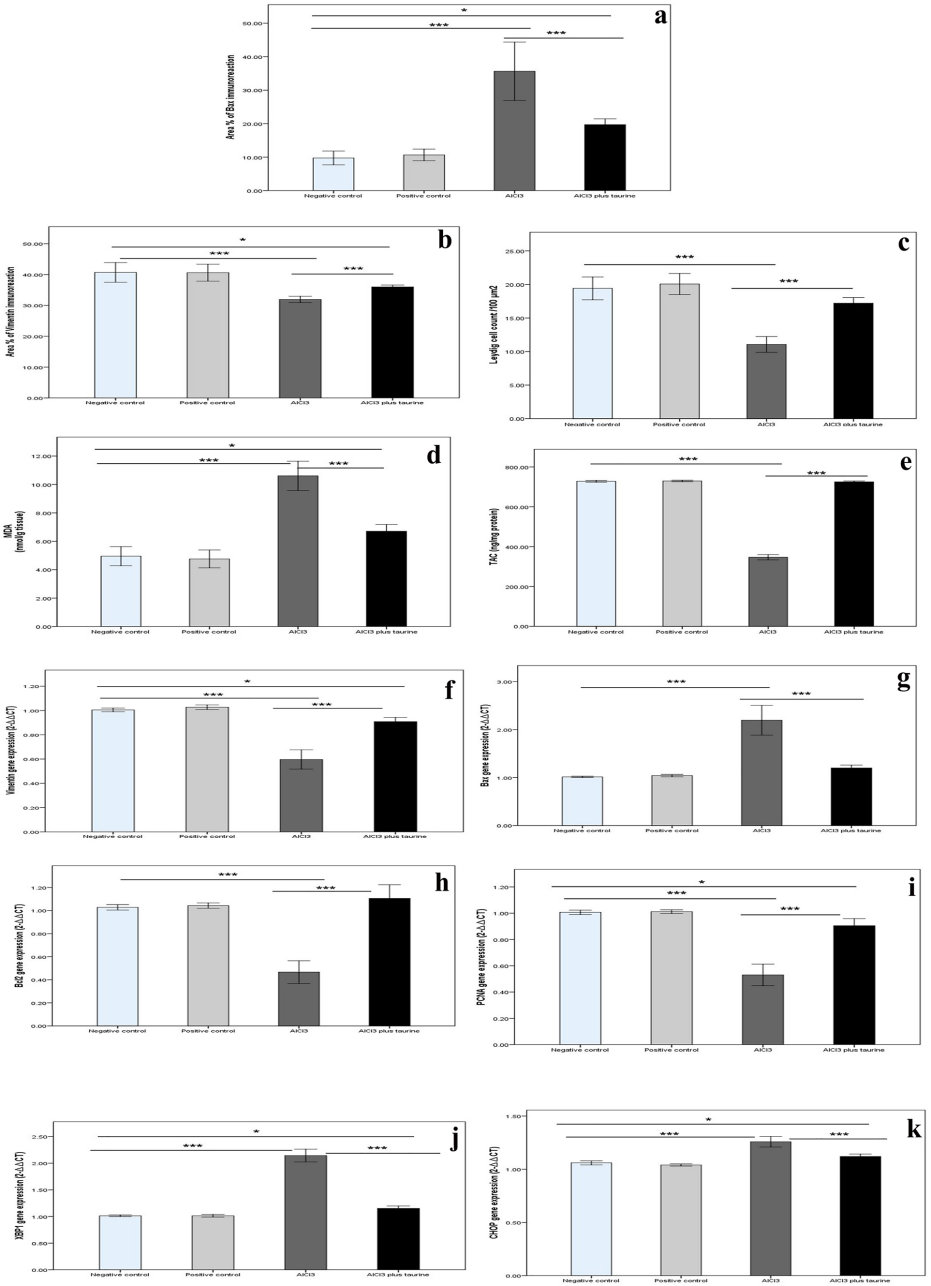
Histomorphomertic, biochemical, and molecular parameters of testes of all studied groups. **a** Area percent of Bax immunoreaction. **b** Area percent of vimentin immunoreaction. **c** Leydig cell (count/100 μm^2^). **d** MDA (nmol/g tissue). **e** TAC (ng/mg protein). **f** *Vimentin* gene expression (2^−∆∆CT^). **g** *Bax* gene expression (2^−∆∆CT^). **h** *Bcl-2* gene expression (2^−∆∆CT^). **i** *PCNA* gene expression (2^−∆∆CT^). **J** *XBP1* gene expression (2^−∆∆CT^). **K** *CHOP* gene expression (2^−∆∆CT^). Data presented as mean ± SD. Statistically significant: *p < 0.05 and ***p < 0.0001

**Fig. 15:**
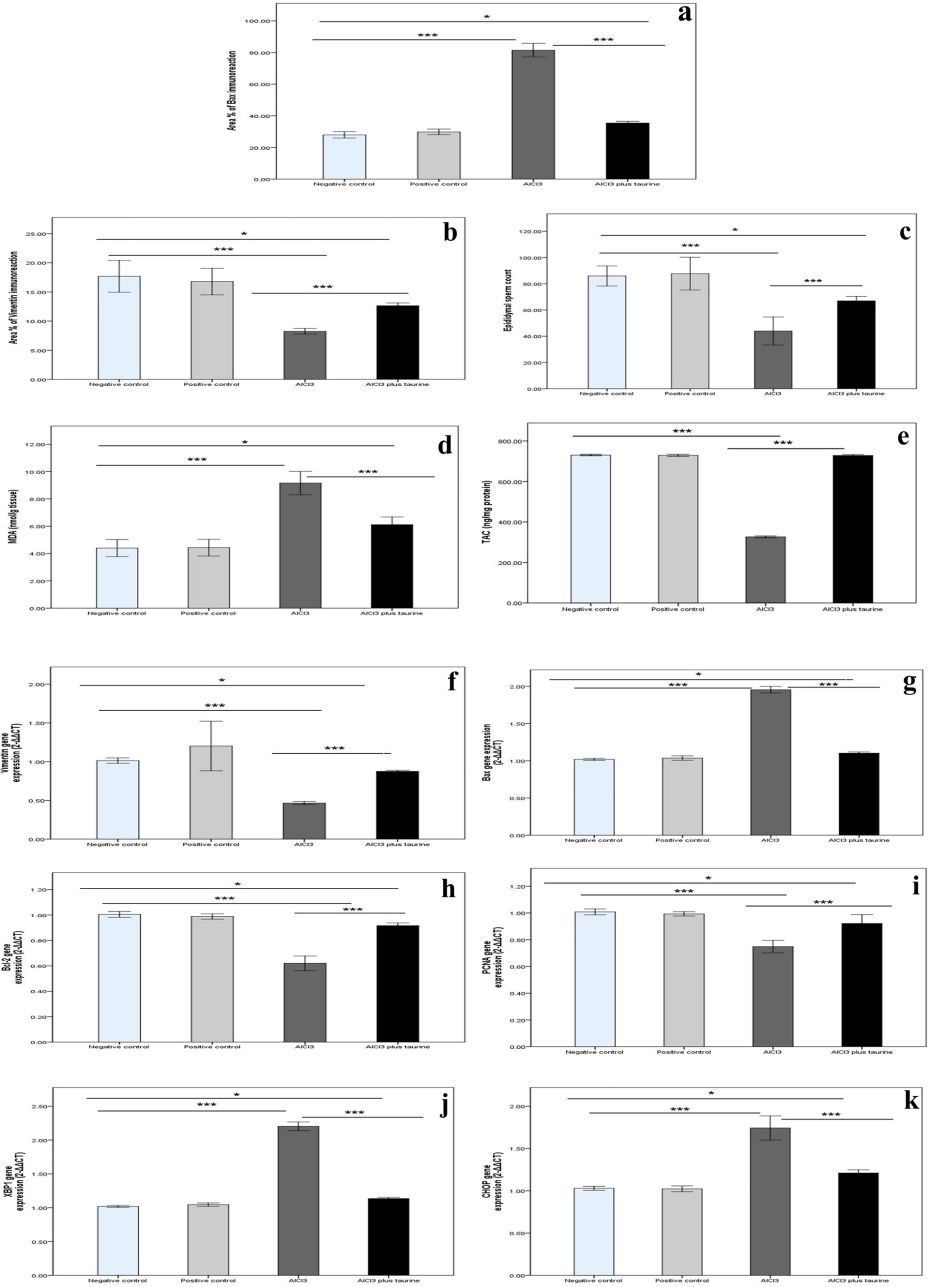
Histomorphomertic, biochemical, and molecular parameters of epididymis of all studied groups. **a** Area percent of Bax immunoreaction. **b** Area percent of vimentin immunoreaction. **c** Epididymal sperm count (million/ml). **d** MDA (nmol/g tissue). **e** TAC (ng/mg protein). **f** *Vimentin* gene expression (2^−∆∆CT^). **g** *Bax* gene expression (2^−∆∆CT^). **h** *Bcl-2* gene expression (2^−∆∆CT^). **i** *PCNA* gene expression (2^−∆∆CT^). **J** *XBP1* gene expression (2^−∆∆CT^). **K** *CHOP* gene expression (2^−∆∆CT^). Data presented as mean ± SD. Statistically significant: *p < 0.05 and ***p < 0.0001

Regarding the area percent of Bax immunoreaction of testis (Fig. 14a) and epididymis (Fig. 15a), a statistically significant increase in the AlCl_3_ group and a non-significant difference in the AlCl_3_ plus taurine group were observed in comparison with control groups. A significant decrease in the area percent of vimentin immunoreaction of both testes (Fig. 14b) and epididymis (Fig. 15b) in the AlCl_3_ group and a non-significant variation in the AlCl_3_ plus taurine group were observed in comparison with control groups. A statistically significant reduction in Leydig cell count (Fig. 14c) and epididymal sperm count (Fig. 15c) in the AlCl_3_ group and a nonsignificant alteration in the AlCl_3_ plus taurine group relative to control groups were detected.

#### Biochemical results

The results of the current study revealed a significant increase in MDA (lipid peroxidation marker) and a significant decrease in TAC in both the testicular (Figs. 14d, 15d) and epididymal (Figs. 14e, 15e) tissues of AlCl_3_ rats compared with control groups. In the AlCl_3_ plus taurine group, MDA was significantly lower than in the AlCl_3_ group but still higher than that of the control groups, in both tissues. Meanwhile, TAC displayed a significant increase relative to the AlCl_3_ group with non-significant changes compared with control groups.

#### Molecular results

In the current study, apoptosis was confirmed at the molecular level by increased *Bax* (Figs. 14g, 15g) and decreased *Bcl-2* (Figs. 14h, 15h) gene expression in AlCl_3_ versus control rats. At the molecular level, decreased *vimentin* expression (Figs. 14f, 15f) was noticed in support of the faint immunoreaction reported at the immunohistochemical level. In addition, a decrease in the cell proliferation factor (*PCNA*) gene expression (Figs. 14i, 15i) was detected in AlCl_3_ compared with control rats. ER stress markers were analyzed at the molecular level by measuring the gene expression of *XBP1* (Figs. 14j, 15j) and *CHOP* (Figs. 14k, 15k), revealing a significant increase in AlCl_3_ compared with control rats.

In the AlCl_3_ plus taurine rats, there was a significant change in the gene expression of the measured parameters compared with the AlCl_3_ group in the form of decreased *CHOP*, *Bax*, and *XBPI* but increased *Bcl-2*, *vimentin*, and *PCNA* gene expression. These results confirm the protective effect of taurine against AlCl_3_-induced male gonadal damage that was detected above at the histological and biochemical levels.

## Discussion

The current study evaluated the harmful effects of AlCl_3_ on the testes and epididymis in rats. Administration of AlCl_3_ to male rats at a dosage of 50 mg/kg/day for 6 weeks led to oxidative stress, reflected as increased testicular and epididymal MDA. These results follow the findings of El-gayar (2016), Yang et al. (2019), and Xu et al. (2020). Lipid peroxidation is the fundamental driver of testicular distortion and consequent dysfunction. Testicular tissue is highly vulnerable to the hazardous effect of free radicals and oxidative stress. This is attributable to numerous explanations involving hyperactive meiosis, cell competition for oxygen rate, low oxygen pressure because of weakened vessels, elevated concentrations of unsaturated fatty acids, and incapability to counteract all free radicals (Asadi et al. 2017), which was confirmed in our study by the suppressed gonadal antioxidants represented by decreased TAC.

Exposure to AlCl_3_ led to significant distortion of the testicular architecture, as previously reported by Pandey and Jain (2013) and Nuhair (2015) in AlCl_3_-exposed animals and confirmed by Khafaga (2017) and Akinola et al. (2021). Guo et al. (2006) and Berihu (2015) stated that these changes were due to excessive Al deposition in rat sera, testis, and epididymis.

In our study, the imbalance between oxidants and antioxidants was associated with an upregulation of the proapoptotic *Bax *and downregulation of the antiapoptotic *Bcl-2*. This was evident by increased expression and immunoreactivity of *Bax* concomitant with decreased expression of *Bcl-2* in both testicular and epididymal tissues in the AlCl_3_ group. This imbalance could explain the histological and apoptotic changes observed at the light microscope level in our study. This aligns with the findings of Ahmed and Mohammed (2021).

Moreover, in the AlCl_3_ group, the degenerated spermatogenic cells detached from the basement membrane of STs and the appearance of Sertoli cells resting on thickened irregular basement membrane with myoid cells could be explained by the defective *vimentin* expression and immunoreactivity as also revealed in our study. This could be a result of AlCl_3_-induced oxidative stress. Vimentin is typically expressed in the basal and perinuclear regions of Sertoli cells and then radiates toward the apical cytoplasm, associated with desmosome-like junctions between the Sertoli cells and adjacent germ cells. Vimentin plays a role in anchoring germ cells to seminiferous epithelium and maintaining the process of proper spermatogenesis through their dissemination in the Sertoli cells via augmenting the contractions of the STs with subsequent transport of spermatozoa and testicular fluid (Nguyen et al. 2019). Diminished *vimentin* expression and immunoreactivity mirror the associated damage of vimentin filaments, leading to the disintegration of the seminiferous epithelium with subsequent defective spermatogenesis (Fang et al. 2017). Thus, the defective spermatogenesis observed in our study could be due to either the decreased expression of *vimentin* and/or the direct impact of oxidative stress on spermatogenesis or testicular tissues (Alam and Kurohmaru 2014; Heinrich et al. 2020).

AlCl_3_-induced oxidative stress and the accompanying prominent apoptosis could inhibit the proliferation and differentiation of Leydig and Sertoli cells, with a subsequent decrease in sperm density and structural damage of the testes and epididymis (Barati et al. 2020). Our results showed a significant decrease of the Leydig cell count in the AlCl_3_ group compared with the controls, as reported previously by Nna et al. (2017). Moreover, the significant low gene expression of testicular *PCNA* could explain the AlCl_3_-induced spermatogenesis dysfunction in the testis and the lower epididymal sperm count. It has been reported that PCNA is a cell proliferation factor that plays a central role in spermatogenic differentiation and hence spermatogenesis (Panahi et al. 2020).

Furthermore, AlCl_3_-associated mitochondrial damage was a prominent feature in the present study in Sertoli and Leydig cells, rounded spermatids, primary spermatocytes, spermatogonia, and spermatozoa. These findings were in agreement with those of Arab-Nozari et al. (2019) and Skalny et al. (2021).

Mitochondrial damage could result from disruption and collapse of the mitochondrial membrane caused by peroxidation of mitochondrial membrane lipids (Koppers et al. 2008) and oxidation of thiol groups of membrane proteins by the increased ROS (Dera and Abushouk 2015). It has also been reported that AlCl_3_ alters the mitochondrial oxidative phosphorylation and electron transfer chain activity, with consequent increased oxidative stress (Iglesias-González et al. 2017), as observed in our study.

Bcl-2 is located in the outer mitochondrial membrane and is essential for regulation of mitochondrial membrane permeability and energy metabolism (Giménez-Cassina and Danial 2015). Another mechanism is the AlCl_3_-induced disturbed mitochondrial transmembrane potential and opening of mitochondrial permeability transition pores (Liu et al. 2020). This leads to uncontrolled entry of water and solutes into the mitochondria, with consequent mitochondrial swelling and damage upon decreased antiapoptotic protein *Bcl-2* expression (Bonora et al. 2020), as observed in our study. Moreover, Bcl-2 can hinder Bax activation. Bax is a cytosolic protein that could be changed, incorporated into the exterior mitochondrial membrane, and oligomerized. These oligomers might enhance the mitochondrial membrane permeability (Giacomello et al. 2020), resulting in cytochrome *c* release that triggers caspase stimulation and apoptosis (Ibrahim et al. 2019). Thus, the elevated Bax/Bcl-2 ratio is a biomarker of mitochondrial dysfunction (Chiu et al. 2018).

Dysregulation of mitochondrial activity is associated with excessive ROS formation, reduced mitochondrial membrane potential, and ROS-mediated activation of apoptosis signal-regulating kinase 1 (ASK1)/p38 mitogen-activated protein kinase (p38MAPK) (Cheng et al. 2017). So, AlCl_3_ could cause excessive ROS production and oxidative stress induction, which eventually triggers mitochondrial-mediated apoptosis, playing an essential role in the pathogenesis of AlCl_3_-induced male gonadal disorders (Lu et al. 2020; Dutta et al. 2021).

Distorted and dysfunctional spermatozoa mitochondrial morphology with subsequent peroxidative destruction of the midpiece and sperm membrane lipids on exposure to AlCl_3_ contribute to gonadal disorders with diminished fertilization ability of spermatozoa (Jurkowska et al. 2019), as seen in the present study. Besides structural sperm proteins, proteins secreted by the epididymis and cytoplasmic repair enzymes are also damaged by ROS (Chianese and Pierantoni 2021).

Similarly, ER stress was profound in this study following exposure to AlCl_3_, as seen on EM examination with concomitant increased *XBP1 *and *CHOP* gene expression in both testicular and epididymal tissues. This is in agreement with the findings of Okail et al. (2020) in the reproductive tract, Al-Kahtani (2010) in the renal convoluted tubular cells, Abdel-Moneim (2013) in the testis, Alghamdi (2018) in the prefrontal cortex, and Aboelwafa et al. (2020) in the hippocampus.

This abnormal ER morphology with consequent ER dysfunction could be explained by the AlCl_3_-induced cellular oxidative stress triggering accumulation of unfolded proteins in ER, resulting in its dilatation and swelling (Promyo et al. 2020). However, it might be due to a direct effect of oxidative stress-induced ER membrane damage (Skalny et al. 2021) with subsequent activation of an ER-associated protein degradation pathway (ERAD). ERAD is a direct activator of the ER-mediated apoptosis process via downregulation of *Bcl-2* and depletion of cellular antioxidants (Sano and Reed 2013), as presented in our study. Specific inducers mediate ER stress apoptosis, the CHOP transcription factor, and the proapoptotic factor Bax, causing a deficiency of the cellular antioxidant capacity, more oxidative stress, and enhancement of apoptosis, as observed in the current study.

Huo et al. (2004) stated that ER chaperone 78-kDa glucose-regulated protein (Grp 78) is present in spermatocytes, so the ER stress signaling pathway is involved in spermatogenesis and as a signaling mechanism for germ cell apoptosis through stimulation of transcription factor 6 (ATF6), inositol requiring enzyme 1 (IRE1), and protein kinase RNA-activated-like ER kinase (PERK). The activation of PERK and IRE1 pathways principally initiates apoptosis under persistent ER stress. Under ER stress, BiP/Grp78 is titrated away by misfolded proteins, leaving p38MAPK primarily activated by stress-related stimuli. It is mainly associated with germ cell apoptosis. Thus, ER stress plays a role in male gonadal function, posttesticular sperm maturation in the epididymis, and fertility capacity (Karna et al. 2019a, b).

In response to ER stress, IRE1α autophosphorylation activates the expression of an active form of XBP1 with potent transcriptional activity. The active XBP1 provokes expression of UPR/ER stress target genes, which induces the inflammatory cytokine's genes by enhancing Toll-like receptor signaling and promoting differentiation of B lymphocytes and inflammatory response (Kim et al. 2015). In the current study, *XBP1* gene expression increased in the AlCl_3_ group, confirming ER stress in testis and epididymis. Hosseini et al. (2020) reported increased *XBP1* expression in the testis and accessory male gonads in response to varicocele-induced oxidative stress and concomitant ER stress, which was confirmed by our results.

The potential protective and therapeutic efficacy of taurine against AlCl_3_-induced deleterious effects was previously reported by Abdel-Moneim (2013), Saad et al. (2018), Wenting et al. (2014), and Niu et al. (2018) on testes, kidney, brain, and memory impairment, respectively. In the current study, we attempted to explore the possible protective effects and the underlying mechanisms of taurine on testes and epididymis distortion and dysfunction induced by AlCl_3_ administration via damping mitochondrial damage and ER stress.

Taurine significantly restored the architecture of testes, epididymis, spermatogonia, spermatids, and spermatozoa. This could be explained by the antioxidant effect of taurine, emphasized by the decreased MDA and increased TAC seen in the current study. Our results are in accordance with the findings of Adedara et al. (2018). Moreover, Adedara and colleagues reported that taurine considerably increased the testicular and epididymal sperm counts and progressive motility. Besides, taurine restored the marker enzymes of testicular function, specifically acid and alkaline phosphatases and lactate dehydrogenase, and conserved the standard ultrastructural features of the testis and epididymis (Adedara et al. 2018). Taurine induced gene expression of the antioxidant nuclear factor (erythroid-derived 2)-like2 (*Nrf2*), glutathione peroxidase-1 (*GPX-1*), and heme oxygenase-1 (*HO-1*) and enhanced the activities of glutathione (GSH) and GPX, resulting in a decline of the MDA content and improvement of the cellular TAC (Agca et al. 2014; Han et al. 2020).

The antiapoptotic effect of taurine was emphasized by a decrease in *Bax* expression and immunoreactivity with a concomitant increase in *Bcl-2* gene expression in taurine-treated rats in this study. These findings were in agreement with the results of Yang et al. (2015). Sedaghat (2021) stated that taurine exerts its antiapoptotic effect by inhibiting the mitochondrial pathway and reverting the gene expression of *Bax/Bcl-2* to normal, as reported herein. Besides, caspase-3 and caspase-9 were also inhibited by taurine (Madbouly et al. 2021).

Taurine is a component of the mitochondrial tRNAs, as two taurine-containing modified uridines were identified. Taurine-conjugated uridines are linked to the role of taurine as an antioxidant (Jong et al. 2021). The antioxidant effect of taurine is associated with an improvement of the mitochondrial function, as it stimulates the mitochondrial protein synthesis and enhances the activity of the electron transfer chain (Bhattacharjee et al. 2021). Also, taurine regulates the intracellular calcium homeostasis, hence protecting against glutamate-induced mitochondrial damage and cell death (Baliou et al. 2021), in addition to its role in the inhibition of mitochondria-mediated apoptosis (Jong et al. 2017), as discussed above.

Taurine prevents the abnormal increase in the mitochondrial calcium level, inhibits the mitochondrial membrane depolarization, protects against the mitochondrial dysfunction and cell damage (Jong et al. 2021), and regulates the energy metabolism (Lan et al. 2021). Moreover, taurine is capable of improving ER homeostasis, and limiting the inflammatory reaction (Stacchiotti et al. 2018). Taurine reduces the apoptosis induced by oxidative free radicals related to ER stress, as it regulates the expression of *XBP1* and other related ER stress genes such as *ATF4* and *CHOP* (Men et al. 2010). These findings are confirmed in the current study by the significant decrease in *XBP1* and *CHOP* gene expression. Taurine also suppresses two of the three UPR pathways as it can improve protein folding by reducing oxidative stress and providing a better osmotic environment for protein folding (Schaffer and Kim 2018).

These taurine-specific mechanisms, together with their detection in the seminal fluid, gonadal vascular endothelial, germinal, and Leydig cells, besides the covering epithelium of efferent gonadal ducts (Rezaee-Tazangi et al. 2020), prove it to be a candidate for protection of gonads from AlCl_3_-induced damage. Moreover, Rezaee-Tazangi et al. (2020) reported that taurine improved the viability, motility, and progressive movement velocity of sperm by suppressing cell death signaling.

Also, taurine displayed a proliferative-inducing effect on testes and epididymis, as confirmed by the highly expressed *PCNA*. These results supported the findings of Liu et al. (2015), who stated that antenatal taurine supplementation increased *PCNA* mRNA expression and the markedly increased PCNA-positive cell counts in intrauterine growth restriction of fetal rat brain tissues improved neuronal regeneration.

In conclusion, oxidative-stress-induced mitochondrial injury and ER stress were fundamental pathophysiological mechanisms that could explain the damaging effect of AlCl_3_ on male gonads. This effect was confirmed by the evident ultrastructural impairment visualized by light and EM examinations of both testicular and epididymal tissues. These findings could be explained by the disturbed gene expression of apoptotic and ER stress markers at the molecular level and the increased oxidative stress markers at the biochemical level. Moreover, taurine supplementation was associated with marked improvement of oxidative stress, mitochondrial injury, ER stress, and apoptotic markers, as evident from the restoration of the standard architecture of both testicular and epididymal tissues. Overall, the results of this study highlight the beneficial effects and mechanisms of action of taurine in the reproductive system of an AlCl_3_-induced gonadal damage rat model. Accordingly, taurine supplementation may be a hopeful natural therapeutic regimen with fewer adverse effects that could be used to inhibit or counteract male reproductive insufficiency.

## Data Availability

Raw data related to the current study are available from the corresponding author upon reasonable request.
